# The potential spread of highly pathogenic avian influenza virus via dynamic contacts between poultry premises in Great Britain

**DOI:** 10.1186/1746-6148-7-59

**Published:** 2011-10-13

**Authors:** Jennifer E Dent, Istvan Z Kiss, Rowland R Kao, Mark Arnold

**Affiliations:** 1 Department of Mathematics and Statistics, 16 Richmond Street, University of Strathclyde, Glasgow, G1 1XQ, UK; 2 Animal Health and Veterinary Laboratories Agency, New Haw, Addlestone, Surrey, KT15 3NB, UK; 3 Department of Mathematics, Mantell Building, University of Sussex, Falmer, Brighton, BN1 9RF, UK; 4 Faculty of Veterinary Medicine, 464 Bearsden Road, Glasgow, G61 1QH, UK; 5 Clinical Trials Research Unit, University of Leeds, Leeds, LS2 9JT, UK

## Abstract

**Background:**

Highly pathogenic avian influenza (HPAI) viruses have had devastating effects on poultry industries worldwide, and there is concern about the potential for HPAI outbreaks in the poultry industry in Great Britain (GB). Critical to the potential for HPAI to spread between poultry premises are the connections made between farms by movements related to human activity. Movement records of catching teams and slaughterhouse vehicles were obtained from a large catching company, and these data were used in a simulation model of HPAI spread between farms serviced by the catching company, and surrounding (geographic) areas. The spread of HPAI through real-time movements was modelled, with the addition of spread via company personnel and local transmission.

**Results:**

The model predicted that although large outbreaks are rare, they may occur, with long distances between infected premises. Final outbreak size was most sensitive to the probability of spread via slaughterhouse-linked movements whereas the probability of onward spread beyond an index premises was most sensitive to the frequency of company personnel movements.

**Conclusions:**

Results obtained from this study show that, whilst there is the possibility that HPAI virus will jump from one cluster of farms to another, movements made by catching teams connected fewer poultry premises in an outbreak situation than slaughterhouses and company personnel. The potential connection of a large number of infected farms, however, highlights the importance of retaining up-to-date data on poultry premises so that control measures can be effectively prioritised in an outbreak situation.

## Background

For a wide range of epidemic infections, contact structures can be used to describe the potential transmission of infection in a population [1-4]. The validity of such models, however, depends on the parameterisation of the contact structures analysed. The existence of the animal movement licensing scheme and cattle tracing system in Great Britain (GB), for which the identification and movements of cattle, sheep, goats, pigs, deer and horses, must be recorded [5], allows for the reconstruction and analysis of the network of contacts in order to predict the spread of infectious disease across these industries. This is not the case for the poultry industry where, before 2005, there was no national register of poultry farms. Motivated by numerous outbreaks of H5N1 highly pathogenic avian influenza (HPAI) across the world and the occurrence of several incursions of avian influenza viruses (AIV) in GB [6,7], information was collected by the British government on poultry farm locations, and on the frequency and types of movements between farms.

In GB, the poultry industry can be divided into the primary breeding sector and the production sector. The biosecurity levels in the primary breeding sector are considered to be consistently high, making the probability of introduction of pathogens into this sector extremely low. In the production sector, birds are purchased from a primary breeding company when they are one day old. Birds then remain on specialist rearing farms until approximately eighteen weeks of age before they are moved to production farms or to hatcheries. Before meat birds enter the food chain, a catching company may be brought in to assist in the catching of birds to be sent to slaughter. Some catching companies may operate on multiple independently owned farms, and some farms may not use a catching company at all, choosing to send birds directly to the slaughterhouse. Vehicles used to transport birds between farms and slaughterhouses are often owned by the slaughterhouse and therefore may act as a link between different production farms. Partly due to the increase in the number and types of movements made on to and off production farms and partly due to increased exposure of birds to the environment in the production sector, it is here where diseases such as AIV have the opportunity to enter a farm, rendering the production sector the focus of this study.

It has already been shown that AIVs have the potential to be spread to a large number of poultry premises via movement of humans and fomites [8,9]. Company personnel, feed lorries, egg collectors, slaughterhouse and catching company personnel (and equipment) have been identified as contact mechanisms between farms, over which disease may transmit [10]. It has also been shown [10] that up to 97%, 42% and 11% of premises associated with slaughterhouses, catching companies and multi-site companies, respectively, are connected. However, this result was reached on the assumption that all premises using the same slaughterhouse, catching company or belonging to the same multi-site company are all potentially connected, with all links being undirected. While most models assume that all potentially infectious connections are always "active" (though see also [1]), in practice there are other factors that will limit the dissemination of disease across the commercial poultry industry. First, over the time that a premises might be expected to be able to transfer infection to other premises, the number of actual connections will depend on the frequency at which the contacts are made. Furthermore, there are likely to be important distance constraints on how far people, vehicles and livestock will travel between premises. Therefore the range over which infection is likely to travel via these means will be limited (although there is currently no maximum journey time for poultry [11]). Also, for catching companies and company personnel, it is possible that there are regional divisions within the company, e.g. geographical sub-divisions within multi-site companies and area-based teams for catching companies, and this has not previously been considered in models of HPAI transmission in GB. Therefore, obtaining detailed data on catching company and slaughterhouse movements for simulation modelling of HPAI in GB has been highlighted as necessary for realistic modelling of HPAI transmission [10]. The objective of this study was to collect detailed movement data from a large catching company and to further explore the potential for HPAI to spread via the routes highlighted.

Based on these new data, an individual farm-based transmission model was developed where nodes are poultry premises with links representing potential transmission routes between premises. Although the static approach (assuming all links between farms are potentially active) that we have previously adopted [10] was appropriate in the absence of detailed link data, the nature of these newly collated data enables us to use a dynamic network model, as is standard practice in disease modelling [2,12], to determine if the frequency and size (distance travelled) of movements between farms is of such a nature as to reduce the concern for a large HPAI epidemic in GB. In this updated model, the presence of links between premises is drawn from the collected movement data. This model also incorporates link directionality allowing for a more realistic and accurate model. Here, the potential for an HPAI epidemic is determined by considering the results of the individual-based transmission model.

## Methods

### Data sources

**Figure 1:**
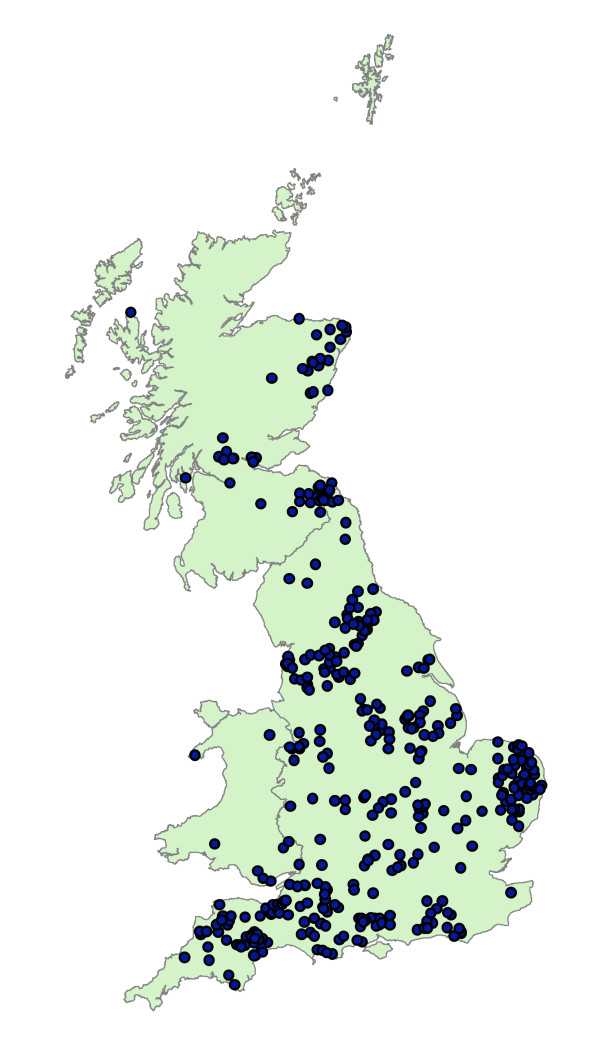
**Distribution of catching company farms in GB**. Map to show the distribution of poultry premises associated with the catching company studied. Each point represents a poultry farm.

Movement data from a major catching company were obtained for all movements made over the 32 month period between 02/01/2005 and 11/08/2007. These data contain the times, dates and premises details for approximately 55,500 movements associated with 68 catching teams (within the company) over 415 poultry premises in GB. The premises associated with this catching company are distributed across GB, as shown in Figure 1. Given that if a premises is visited by a catching team, then there must be a movement on the same day between the premises and a slaughterhouse, these movement data also include movements of birds from premises to slaughter.

Population data on all commercial poultry premises housing 50 or more birds, and within 15 km of farms visited by teams belonging to the catching company, were taken in November 2007 from an extract of the GB Poultry Register (GBPR), provided by the Department for environment, food and rural affairs (Defra). The addition of these premises allowed us to consider disease transmission that can occur between premises that are in close spatial proximity, enabling us to consider how likely infection is to jump from the network of premises for which we have movement data to another network of premises for which no movement data were available for analysis. The addition of these premises brought the number of premises studied up to 10,692. The total number of premises recorded in the GBPR extract studied was 24,075. Although the total number of premises serviced by the catching company is small (approximately 2% of all premises in the GBPR), by comparing the number of premises serviced by the catching company with the number of premises known to be associated with the largest catching companies in England and Wales, we estimate that between 30% and 50% of premises serviced by a major contracted catching company are accounted for in this dataset [13 and unpublished data]. No data were available for small, independent catching companies.

Premises in the catching company database were matched to premises in the GBPR so that the model could be parameterised using location, premises population size and species data. Temporal aspects were accounted for by preserving the order in which poultry premises were visited by individual catching teams on a daily basis. Additional information on which premises belong to integrated companies was obtained from a sample survey of integrated companies.

In the absence of quantitative movement data of company personnel for the farms studied, we sought expert opinion [P. Mcmullin, Poultry Health Services, *pers. comm*.] to inform the model of the likely frequency of movements of personnel between farms, based on species type, farm size and distance between farms belonging to the same company. The probability of companies using staff on multiple farms was estimated as well as the estimated frequency of visits of veterinary officials and area managers to poultry premises (Table 1).

**Table 1:** Model parameters.

Parameter	Value	Source
Incubation period	Up to 1 day (shedding after 8 hours, death after one day)	I. Brown (*pers. comm*); [14,15]
Survival of virus on seed premises	Up to15 days	[16]
Probability of staff working on multiple premises (Farm size)	0.45 (< 50,000 birds) 0.1 (50,000 to 200,000 birds) 0 (> 200,000 birds)	P. McMullin (*pers. comm*)
Distance travelled by staff between premises	Up to 35 km	P McMullin (*pers. comm*.)
Frequency of vet visits	Every 50 days	P. McMullin (*pers. comm*)
Frequency of manager visits	Every 10 days (non-layer farms) Every 50 days (layer farms)	P. McMullin (*pers. comm*)
Probability catching team catches species (multi species farms only)	0.7 Chicken 0.12 Turkey 0.16 Duck/goose 0.01 Other	Calculated from catching company data, where species type available
Probability of catching team, slaughterhouse and owner transmission	0 to 0.2, in steps of 0.01, with additional parameter at 0.001 added.	N/A
Time to detection	2 to 6 days (15 days later for ducks/geese)	Extrapolation from [15-17]

Parameter values for the network simulation model of avian influenza transmission in Great Britain

### Descriptive analysis

A descriptive analysis of the collected data is given in Additional File 1. This analysis was used to identify any trends in the data that could have important implications for disease transmission. In particular we considered how far catching teams and slaughterhouse vehicles travelled between premises, as it was shown in [10] that this had a large impact on the potential size of an HPAI outbreak. The frequency of catching team movements to premises, dependent on farm size, was also considered in order to determine if premises size should be recorded as an output of the simulation model.

### Simulation model (see also Additional File 2)

A stochastic simulation model at the farm level was developed where farms were classed as susceptible, infected, detected or culled. HPAI could be transmitted between premises in close spatial proximity or through contact via catching teams, slaughterhouse vehicles or personnel movements within an integrated (multi-site) company. A random number generator chooses a premises in the network to infect and a random date of infection (within the 886 days covered by the catching company data set). If the seed farm was not visited within 15 days of this time point, assumed to be the maximum time that HPAI would survive in the farm environment, then transmission will be limited to local spread, often resulting in little or no onward transmission from the seed premises. When transmission beyond the seed premises did occur, outbreaks were allowed to run their course.

We assumed a time step of one day in the model, so that for each day of the simulation, once a premises had become infected, we assumed silent spread up to the time of detection. Detection and culling dates were set within the model at time of infection and were dependent on whether the infected premises was in a protection zone (PZ), a surveillance zone (SZ), or neither, as described below.

On detection of notifiable HPAI in poultry, 10 km SZs and 3 km PZs are typically set up around infected premises. In the model, we assumed no transmission within the PZ/SZ via the normal movement of catching companies or slaughterhouse equipment since all movements in those zones would be monitored. Therefore, spread would only continue within the PZ/SZ via local spread. Time to detection within an infected flock was assumed to occur between 2 and 6 days (mean at 4 days) after infection [14-18], with the mean reduced to 3 days for flocks within a PZ or SZ. Culling occurs at the end of an epidemic day. Using the time taken to cull birds in the most recent outbreak of HPAI H5N1 in GB [19], we assumed that culling was completed on infected farms within 3 days of detection. One hundred simulations were run for a range of parameter values (Table 1). Up to 1,000 simulations were run for a subset of parameter combinations with no qualitative change in the results. For each simulation, the times at which each farm had become infected and the times at which their state changed from infected to detected and from detected to culled was recorded.

#### Catching team and slaughterhouse movements

When a movement occurred between infected and susceptible farms, infection was spread between farms with probability relating to the type of movement made. Where multiple species were held on one farm, we assumed that catching teams catch, on average, one species per visit with probability defined in Table 1. This species became infected with probability of transmission via catching team. If a susceptible farm had multiple links from an infected premises, then each link was treated independently, and the probability of infection therefore increased. We assumed that catching team and slaughterhouse vehicles do not remain infectious overnight, as we assume that effective decontamination procedures were in place. A premises was designated as infected if one or more species on the premises was infected.

#### Company (personnel) movements

We assume that spread of infection between farms belonging to the same multi-site, integrated company could occur either via the movements of area managers or veterinary officials between premises, or via staff working on multiple farms. Movements of veterinary officials, of area managers and of company personnel were simulated on a per day basis by using farm size and distance between farms belonging to the same multi-site company to identify using parameters in Table 1 if a) two premises are to be visited on the same day by the same person and, b) if the link made between premises will result in transmission of disease. The model assumes independence between days so that the last time a premises was visited is not accounted for.

#### Local (spatial) spread (see Additional Files 2 and 3)

Based on expert opinion, we assume spatial (primarily airborne) spread in GB is likely to occur with small probability (p ≤ 0.01) and only for distances up to a maximum 0.5 km [D. Alexander, R. Irvine *pers. comm*.], according to the density kernel that is given in Equation 1.

For dist < 0.5 km:

(1) $$p\left(trans|dist\right)=0.01\;{\left[{\left(1-dist\;/)\;0.5\right)}^{\;2}\right]}^{2}\;$$

*Else p*(*trans|dist*) = 0

A sensitivity analysis of the model to the assumption that airborne spread could occur is given in Additional File 3.

### Analysis of simulation output

We first consider the proportion of outbreaks that resulted in onward spread beyond the seed case. Here, the results follow a linear trend and, as the outcome is a binary variable (essentially secondary spread, or no secondary spread) dependent on explanatory variables that can be categorised into multiple levels, the analysis lends itself to a logistic regression. Thus a binary logistic regression was done (using Minitab v16) on the proportion of outbreaks that resulted in onward spread beyond the seed case. We next consider final epidemic size. In order to determine how the different types of transmission affect the epidemic size, two logistic regression models were fitted (Minitab v16). In the first, the binary response variable describes whether a small (< 25 premises) epidemic occurs or not. In the second, the binary response variable describes whether a large (> 65 premises) epidemic occurs or not. In both cases, the explanatory variables are the simulated transmission probabilities for AIV transmission via catching company, slaughterhouse- and owner-related movements.

## Results

Additional File 1 gives a data summary and descriptive analysis of the catching company data. The key points of this analysis are given below:

1. When the frequency of movements is not accounted for, slaughterhouse related movements connected 94% of premises, catching team movements connected 76% of premises and owner movements 11% of premises that are associated with the catching company. However, when time is considered, catching teams connect only 2 premises per day and slaughterhouses an average of 3 premises per day.

2. Contrary to expectations, the data presented show that premises do use multiple slaughterhouses and are associated with multiple catching teams within the same company (consecutively over the time period studied). There is no overlap between poultry companies (i.e. poultry premises are associated with only one poultry company).

3. There is an increase in frequency of visits to larger premises, implying that these premises will be at a higher risk of infection should infection be transmitted by catching team or slaughterhouse vehicles/equipment.

4. Slaughterhouse vehicles and catching teams travel long distances between premises, with 72% of movements between premises exceeding 10 km in length.

### Simulation modelling

One hundred simulations were run for a total of 10,648 scenarios. Each scenario represents a different combination of transmission values for each of the transmission routes studied. In particular, each scenario was created by ranging parameters from 0 to 0.2, in a step-wise fashion, such that each parameter took on one of 22 possible values within this range, giving rise to *22^3 ^= 10,648 *scenarios.

### Proportion of positive epidemics spread beyond the index case

The aims of the simulation model are to determine if a large outbreak of AIV is possible in the poultry industry in GB and, if so, what might cause a large outbreak to occur. One way of answering the first question is to consider how often infection spreads beyond the seed premises. That is to ask "how many simulated outbreaks result in secondary spread?"

**Figure 2:**
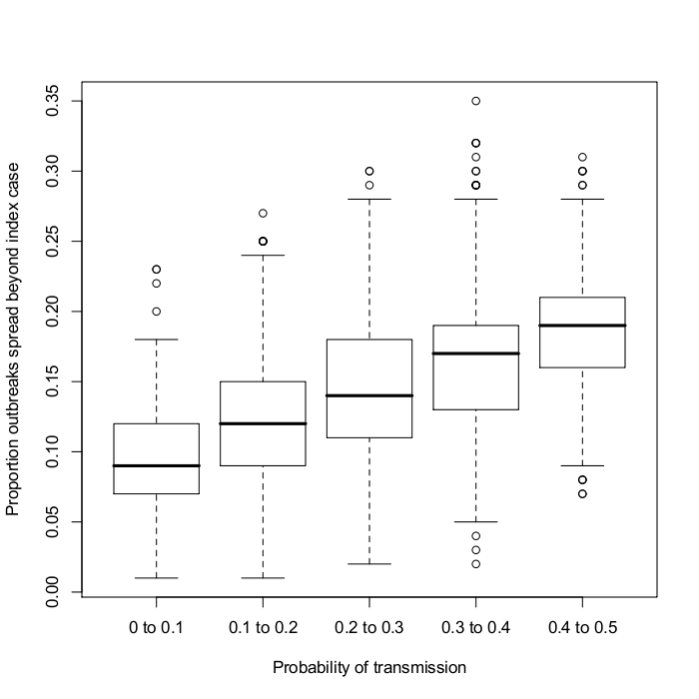
**The proportion of outbreaks that spread beyond the seed premises for all simulation results**. Boxplots to show the median, quartiles and outer points of the proportion of outbreaks (over 100 simulations) that spread beyond the seed premises, for increasing rates of transmission. Here, transmission is recorded as the combined risk of AIV transmission over all routes, according to Equation 2.

When all scenarios and all simulation results are considered together, infection spread beyond the seed premises in approximately 15% of the simulations run (mean value over all simulations and all scenarios). Figure 2 shows how the distribution of infections that result in secondary spread varies as the probability of AIV transmission is increased. For this, the probability of transmission was calculated by combining the probability of transmission via catching team, slaughterhouse and company personnel, as shown in Equation 2.

(2) $$P\left(i\;Infected\right)=1-\left(\overset{3}{\underset{j-1}{\prod }}\left(1-p_{j}\right)\right)$$

for *p_j _*probability of infection for via link type *j*.

Infection resulted in secondary spread (beyond the seed premises) in up to 35% of scenarios. The simulation that gave the maximum number of cases that spread beyond the seed premises was from the following scenario: catching team (cc) = 0.04, company personnel (owner) = 0.19 and slaughterhouse (sh) = 0.13. This suggests that high probabilities of transmission are not necessary in all three potential transmission routes for infection to (relatively) frequently spread beyond the index case.

Results from a logistic regression analysis are shown in Tables 2, 3 and 4. Consider the odds ratios in Tables 2, 3 and 4. The results show that transmission via the movement of catching teams does not have a significant effect on the probability that an outbreak will result in secondary spread. However, movements related to company personal appear to be significant at all levels, with the exception of transmission set to *p = 0.001*. Movements related to slaughterhouse vehicles are significant in the probability that an outbreak will result in onward spread only when the probability of transmission is high enough (here, the model predicts a rate of > 0.06 for a significant effect to be seen). Further, the odds ratios also tell us that, under the assumptions made, as the rate of transmission increases for owner movements in particular, the effect on the probability of secondary spread is increasingly large, with the odds ratio rising to 2.11 (2.03, 2.19 (95% CIs)) for a transmission rate of 0.14 compared to zero. This suggests that the probability of secondary spread beyond the seed premises is not uniformly affected by transmission rates across the different link types. This is driven by the characteristics of the networks over which disease can spread.

**Table 2:** Binary logistic regression, with odds ratios calculated for the probability of secondary spread versus catching company transmission rates

Transmission rate	Odds Ratio	Lower 95% CI	Upper 95% CI	p-value
0.001	0.97	0.93	1	0.064

0.01	1	0.97	1.04	0.813

0.02	0.95	0.92	0.98	0.005

0.03	0.97	0.94	1.01	0.14

0.04	1.02	0.98	1.06	0.303

0.05	0.96	0.93	0.99	0.024

0.06	0.96	0.93	1	0.043

0.07	0.99	0.95	1.02	0.451

0.08	0.96	0.93	1	0.038

0.09	0.95	0.92	0.99	0.011

0.1	0.97	0.94	1.01	0.124

0.11	0.98	0.94	1.01	0.23

0.12	0.99	0.96	1.03	0.677

0.13	1	0.97	1.04	0.906

0.14	0.98	0.94	1.01	0.187

0.15	0.98	0.95	1.02	0.267

0.16	0.96	0.92	0.99	0.018

0.17	1	0.96	1.03	0.871

0.18	0.98	0.95	1.02	0.313

0.19	0.96	0.93	1	0.047

0.2	0.98	0.95	1.02	0.359

**Table 3:** Binary logistic regression, with odds ratios calculated for the probability of secondary spread versus owner transmission rates

Transmission rate	Odds Ratio	Lower 95% CI	Upper 95% CI	p-value
0.001	0.98	0.94	1.03	0.488

0.01	1.09	1.04	1.14	0

0.02	1.19	1.14	1.24	0

0.03	1.27	1.22	1.33	0

0.04	1.38	1.33	1.44	0

0.05	1.42	1.37	1.48	0

0.06	1.47	1.41	1.53	0

0.07	1.53	1.47	1.59	0

0.08	1.66	1.59	1.72	0

0.09	1.74	1.67	1.81	0

0.1	1.75	1.69	1.83	0

0.11	1.86	1.79	1.94	0

0.12	1.97	1.89	2.04	0

0.13	1.94	1.87	2.02	0

0.14	2.11	2.03	2.19	0

0.15	2.09	2.01	2.17	0

0.16	2.19	2.11	2.27	0

0.17	2.24	2.16	2.33	0

0.18	2.33	2.24	2.42	0

0.19	2.38	2.29	2.47	0

0.2	2.38	2.29	2.47	0

**Table 4:** Binary logistic regression, with odds ratios calculated for the probability of secondary spread versus slaughterhouse transmission rates

Transmission rate	Odds Ratio	Lower 95% CI	Upper 95% CI	p-value
0.001	0.97	0.93	1.01	0.093

0.01	0.99	0.95	1.02	0.435

0.02	0.99	0.95	1.03	0.597

0.03	1	0.96	1.03	0.861

0.04	1	0.96	1.03	0.824

0.05	1	0.96	1.04	0.927

0.06	1.04	1	1.08	0.036

0.07	1.03	1	1.07	0.068

0.08	1.04	1.01	1.08	0.019

0.09	1.04	1.01	1.08	0.019

0.1	1.05	1.01	1.09	0.012

0.11	1.07	1.03	1.11	0

0.12	1.05	1.01	1.09	0.008

0.13	1.09	1.05	1.13	0

0.14	1.08	1.04	1.12	0

0.15	1.09	1.06	1.13	0

0.16	1.08	1.04	1.12	0

0.17	1.08	1.04	1.12	0

0.18	1.09	1.06	1.13	0

0.19	1.1	1.06	1.14	0

0.2	1.1	1.06	1.14	0

**Figure 3:**
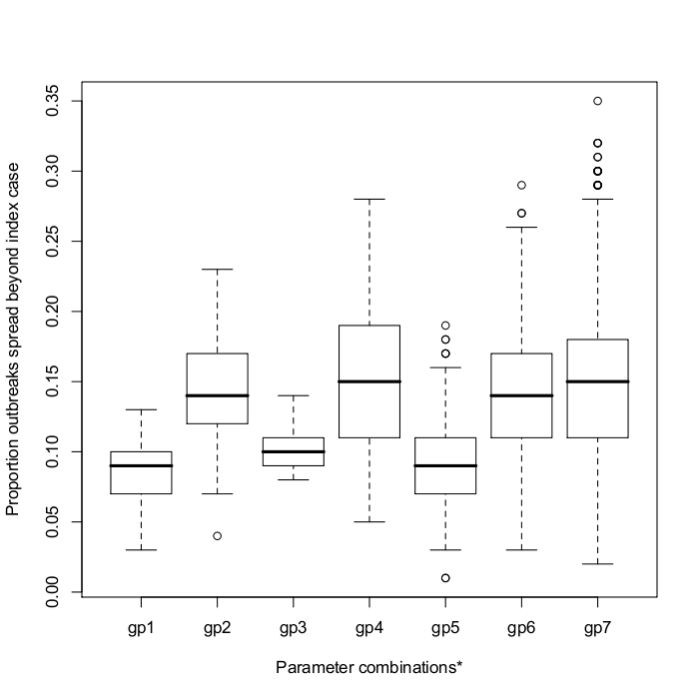
**The proportion of outbreaks that spread beyond the seed premises for different parameter combinations**. Boxplots of the proportion of outbreaks that result in spread beyond the seed premises, for different parameter combinations. gp1 = sh, gp2 = owner, gp3 = cc, gp4 = owner and sh, gp5 = cc and sh, gp6 = cc and owner, gp7 = cc, owner and sh. Within each group, parameters are varied from 0 to 0.2.

In order to visualise the effect that the interaction of different transmission routes can have on the results, each potential transmission route was considered on its own as well as in combination with one or more other potential routes of transmission. Figure 3 shows boxplots that describe the proportion of outbreaks that result in onward spread for different scenarios. The figure shows that a higher proportion of outbreaks occur for transmission via owner-related (gp2) movements than for catching company (gp1) or slaughterhouse-related (gp3) movements. It appears that adding catching company transmission to either transmission via slaughterhouse- or owner-related movements (gp5 and gp6, respectively) has little impact on the proportion of outbreaks that would result in secondary spread if the main effects were considered alone. However, the combination of slaughterhouse- and owner-related movements (gp4) suggests that this combination can result in a large proportion of outbreaks resulting in secondary spread. Finally, it is interesting to note that Figure 3 also shows that when all three transmission routes (cc, sh and owner) are greater than zero, a large proportion of outbreaks can result in onward spread (gp7).

The statistical significance of interaction terms can be determined by refitting the logistic regression model, with interaction terms included. As the model did not converge when all tested transmission rates were considered as a single level, in order to consider the potential interaction between different networks the data were categorised into "high", "medium" and "low" probabilities of transmission and the model refitted (see Additional File 4 Table S1). Although the results (Additional File 4 Table S1) show that only medium and high levels of owner transmission have a significant effect on the results, for all levels of owner/slaughterhouse interaction, there was a significant difference between the results from this interaction, compared to zero. This implies that whilst slaughterhouse transmission alone is not enough for an outbreak to result in secondary spread, the combination of owner and slaughterhouse related movements has a significant effect on the probability that an outbreak results in secondary spread, even for low levels of transmission of disease. The results from Additional File 4 Table S1 also show that there is no significant interaction effect from the catching company - owner interaction or from the catching company - slaughterhouse interaction (the confidence intervals on all odds ratio include zero). This means that, in theory, catching company transmission can be dropped from the model. With catching company removed from the results and the regression rerun, the final logistic regression results are given in Table 5.

**Table 5:** Binary Logistic regression: secondary spread versus transmission rates for interaction between transmission routes at different levels of transmission.

Predictor	Coefficient	SE	Odds Ratio	Lower 95% CI	Upper 95% CI	p-value
Constant	-2.16587	0.071836				0

owncat						

1	0.004215	0.076562	1	0.86	1.17	0.956

2	0.415397	0.075335	1.51	1.31	1.76	0

3	0.631829	0.074869	1.88	1.62	2.18	0

shcat						

1	-0.22299	0.077486	0.8	0.69	0.93	0.004

2	-0.12265	0.077053	0.88	0.76	1.03	0.111

3	-0.0784	0.076875	0.92	0.8	1.07	0.308

owncat*shcat						

1*1	0.248773	0.082484	1.28	1.09	1.51	0.003

1*2	0.227419	0.082041	1.26	1.07	1.47	0.006

1*3	0.229411	0.081855	1.26	1.07	1.48	0.005

2*1	0.207304	0.0812	1.23	1.05	1.44	0.011

2*2	0.154659	0.080772	1.17	1	1.37	0.056

2*3	0.140458	0.080593	1.15	0.98	1.35	0.081

3*1	0.228509	0.0807	1.26	1.07	1.47	0.005

3*2	0.174293	0.080272	1.19	1.02	1.39	0.03

3*3	0.15525	0.080095	1.17	1	1.37	0.053

Final model.

Level 1 = low transmission rate 0 - 0.06, level 2 = medium transmission rate 0.07 - 0.13, level 3 = high transmission rate 0.14 - 0.2. sh = slaughterhouse, own = company personnel. SE = standard error.

### Epidemic size

In this part of the analysis, only epidemics that result in spread beyond the seed premises are considered. This accounts for approximately 15% of all simulation results.

**Figure 4:**
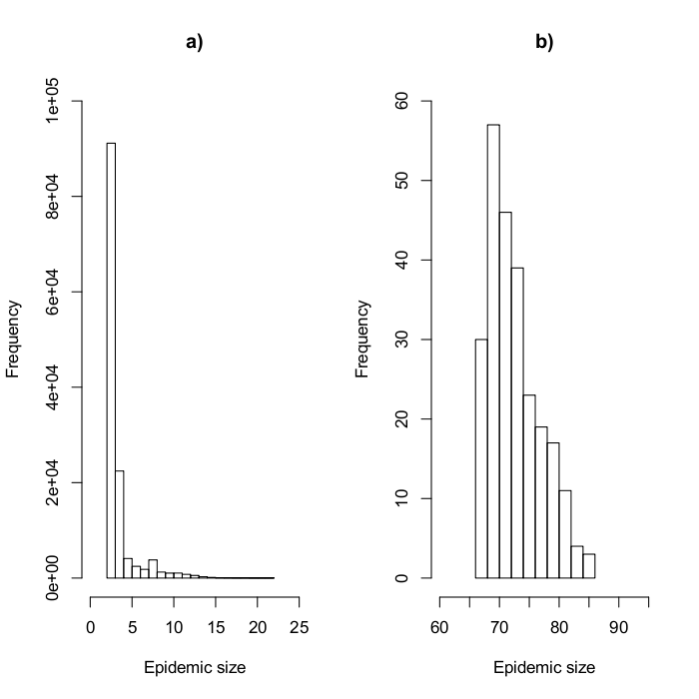
**Epidemic size**. Histogram of epidemic size for infections resulting in onward spread beyond the seed premises. a) epidemics including fewer than 25 infected premises and b) epidemics including more than 65 infected premises.

For all results, there were no epidemics of size between 23 and 66 premises (see Figure 4). The number of large epidemics, which we consider here to involve more than 65 infected premises (those epidemics in Figure 4b), is small, representing 0.2% of all results. However, these are the epidemics that are likely to cause the most strain on resources in an outbreak situation, so it is important to determine from the data if the rate of transmission via different routes, or the index premises in these epidemics, have any notable characteristics.

There were a total of 330 individual premises that were included in the set of outbreaks that resulted in onward spread (~80% of population for which movement data were available). Of these, 95 individual premises were seed premises in the (249) "large" epidemics recorded. All 95 of these premises were also seed premises in the list of (130939) "small" epidemics. Premises size (number of birds) was available for 78% of seed premises for large epidemics, and for 94% of seed premises for small epidemics. The results (Table 6) suggest that a large epidemic is more likely to occur when infection is seeded in large premises (37 of 74 "large" epidemics began in large premises). However, infection into large premises does not imply a large epidemic will occur. Interestingly, the mean epidemic size when infection is seeded in small premises (3.8) is larger than that of both medium (2.8) and large premises (3.1). This may be connected to the probability of an outbreak resulting in spread beyond the seed premises, as owner links have been shown to be important and owner movements are more likely to occur in small premises (an immediate effect of the model assumptions).

**Table 6:** Effect of seed premises on outbreak size.

Seed premises size	Number unique premises in small epidemics (seed)	Number unique premises in large epidemics (seed)	Proportion outbreaks resulting in large epidemics
Small (≤ 100,000 birds)	35	20	0.57

Medium (100,000 - 200,000 birds)	59	17	0.29

Large (> 200,000 birds)	141	37	0.26

The proportion of outbreaks that result in large/small epidemics for different size categories of seed premises.

In order to determine how the different types of transmission affect the epidemic size, two logistic regression models were fitted. In the first, the binary response variable describes whether a small (< 25 premises) epidemic occurs or not. In the second, the binary response variable describes whether a large (> 65 premises) epidemic occurs or not. In both cases, the explanatory variables are the simulated transmission probabilities for AIV transmission via catching company, slaughterhouse- and owner-related movements. The results are shown in Additional File 4 Table S2 to S7.

For small epidemics, Additional File 4 Tables S2 to S4 show that catching company movements have a significant influence on the results for a range of probability values between 0.02 and 0.16. Interestingly, when these results are significant (the odds ratio confidence intervals do not contain zero), the odds ratios show that the probability of a small epidemic decreases (the odds ratios are less than 1) with an increase in catching company transmission rates, when compared to zero. This suggests that an increase in catching company transmission might result in a higher proportion of epidemics being larger. Additional File 4 Table S3 shows that transmission via owner movements is significant at all levels above *p = 0.001*. Above this value, the odds ratios are all larger than one, implying that increasing the rate of transmission results in the likelihood of a small epidemic occurring to increase. For slaughterhouses (Additional File 4 Table S4), significant results are obtained for transmission rates > 0.05. The strength of the significance does not increase in proportion with the increase in transmission, with all transmission rates > 0.12 having an odds ratio value of between 1.07 and 1.10. These results therefore suggest that the most influential parameter for the probability of a small epidemic to occur is transmission via owner movements.

Additional File 4 Table S5 and S6 show that, contrary to expectations, neither catching company nor owner movements play a significant role in the probability that an outbreak will result in a large epidemic. For large epidemics, the most influential predictor is the transmission rate via slaughterhouse linked movemernts (Additional File 4 Table S7). Analysis of the odds ratios for slaughterhouse transmission versus large epidemics shows that this transmission route is only influential if it is high enough (above 0.12). However, when it is high enough, the upper 95% limits (for the odds ratios) show that an increase from zero transmission to a higher transmission rate will result in a large epidemic being up to 28 times more likely. This is a very striking result with heavy implications on resources, for example, in the event of an outbreak. It is therefore essential to determine the true probability of transmission via this route.

### Spatial spread

While the majority of outbreaks did not result in further onward transmission from the index premises, outbreaks could potentially cover up to 20% of the population (for a range of parameter values < 0.2) for which network data were available, covering distances of up to 730 km between premises (see Additional File 5, Figure S1). Although for the largest distances to be covered (> 700 km), at least one transmission parameter must be as high as 0.12, occasionally, distances over 600 km between premises are reached for transmission parameters between zero and 0.07. This has important implications for the availability of control resources as the number of premises in the SZs will be greater if dissemination of virus is geographically widespread and therefore potentially involving a larger number of local disease control centres. Large epidemics invariably resulted in widespread geographical dissemination of virus.

In this model, infection can only be spread into premises that are not serviced by the catching company, by spatial transmission of disease, to premises within 500 m of infected premises. This results in infection of premises that are potentially connected to different sub-networks (via other catching companies, slaughterhouses or poultry companies for example) in less than 1% of the simulations run. However, we have seen that transmission via sh-linked movements is an important factor in determining final epidemic size, and slaughterhouses that are included in the network studied may also be used by poultry premises not included here. This implies that if this route is important, infection may leak into other sub-networks of the industry much more frequently.

## Discussion

Despite the extent of data previously available on the British poultry industry, the detailed contact structures within the poultry industry in GB have only been poorly understood. Previous studies have been able to identify potential contact structures but assumptions have had to be made on the frequency and patterns of movements between farms [10,15,20]. Whilst it is important to acknowledge that the models presented here rely heavily on expert opinion (which is arguably a drawback of such a modelling approach), in the absence of outbreak data for AIV in GB, this cannot be avoided. For this reason, we have considered many scenarios by varying parameter values and by combining expert opinion with real time movement data from a large catching company, we have been able to adopt a similar approach to that used in [1] to investigate the potential spread of AIV in the GB poultry industry.

The results presented here show that restrictions on the frequency of movements can have an important role in determining disease spread risk. In particular, connections via slaughterhouses can connect a large number of premises over a large geographical area, important in the potential for virus dissemination. Spread via slaughterhouse-linked movements is most prominent when partial flock depopulation is being undertaken at a farm, as this action results in more premises being visited in one day and potential infection of birds that remain on the farm. This is also an important output for the control of diseases other than HPAI, such as *Salmonella *or Campylobacter spp., where the slaughterhouse is a more likely reservoir for pathogens [21]. We note here that, whilst slaughterhouses and catching teams are separated in this study, in some cases one might group the two transmission routes together under the assumption that any movement that arises due to a catching team visiting a farm is considered a 'catching company' movement. The results are likely to be sensitive to such an assumption and thus it is important not to misinterpret them. However, the principles used in this study remain valid for the potential transmission of diseases spread by the faeco-oral route, such as Campylobacter spp. and *Salmonella*, as well as different strains of HPAI. The model is well-suited to investigating diseases where expert opinion does not have to be so heavily relied upon for model parameterisation (as expert opinion adds uncertainty to results, resulting in one only being able to answer 'what-if' scenarios, in a situation the model assumptions may affect interpretation of results). In Campylobacter research, for example, one would expect the model results to be quite different due to difference in epidemiological characteristics of Campylobacter spp. compared to HPAI. With a perceived higher prevalence of the pathogen, we would expect the results of the model presented here, applied to Campylobacter spp. to show that catching company movements are likely to have a bigger effect on the spread of disease between farms.

Despite the relatively heavy use of expert opinion to estimate model parameters in this study - in particular for the frequency of movements made by company personnel, we can use the model presented here to hypothesise about the importance of different types of potentially infectious links between poultry premises and we can conclude from these results that, where slaughterhouses can act as a reservoir for pathogens, the spread via this route should be minimized. This can be achieved through additional bio-security measures, such as thorough cleaning of the crates and vehicles that carry the birds, for example.

The results that catching team movements have little effect both on the probability of an outbreak resulting in onward spread beyond the seed premises and on the probability of a large epidemic occurring are important results, as they suggest that the number of farms that a catching team visits during the infectious period of the virus is too low to link a high number of farms, in GB, during an epidemic. For pathogens that can survive for longer periods in the environment or that are more prevalent than HPAI (such as Campylobacter spp.), the number of farms that can be linked by catching team movements will be (potentially significantly) higher. However, while extensive and therefore of value, the data used here correspond to only one (large) catching company that is made up of a 68 distinct catching teams. As each farm may be visited by one or more of the catching teams, there are no distinct regional divisions apparent within this company as was initially expected. Further, these data do not consider further spread once other networks (e.g. connected by slaughterhouses and catching companies) contain infected premises.

Although all three transmission routes were positive when a large proportion of (simulated) outbreaks resulted in spread beyond the seed premises, the fitting of a regression models suggests that only company personnel movements significantly influence the probability that infection will spread beyond the seed premises. This highlights the importance of obtaining more accurate estimates on the frequency of movements of company personnel and the probability of transmission via this route.

There was a significant interaction effect for the owner*slaughterhouse interaction on the proportion of outbreaks that result in onward spread. However, the combinations of potential transmission of disease via catching company and company personnel movements, or slaughterhouse-linked and catching company movements have little effect on the proportion of outbreaks that result in onward spread, particularly compared to the individual owner effect. This can be explained by the frequency of movements relative to premises size (Additional File 1 Figures S6 to S9), such that the increased frequency of catching company movements in particular (and also, but less so for slaughterhouse-linked movements), to larger premises is not high enough to force these potential transmission routes to have a large effect on the proportion of outbreaks that result in spread beyond the seed premises, compared to transmission via owner movements. Having highlighted owner movements as important in previous studies [10] and given that they can have a large effect on the number of outbreaks resulting in an epidemic, it is recommended that data collection is expanded to include movement data from an integrated company, furthering our ability to provide more robust estimates of epidemic size and likelihood.

The results show that there is a "jump" from epidemics of size lower than 23 infected farms (< 5% of premises), to epidemics containing more that 65 infected farms (~20% of premises). This is in line with results published by [22], who report that a predictor of the need to intensify control efforts in GB is whether an outbreak exceeds 20 infected premises. The results follow the pattern of epidemic outbreak sizes (at least qualitatively) as expected for any stochastic epidemic model, with epidemics either going extinct early, or growing to reach a substantial proportion of the population. Whilst this result, which represents a threshold for the basic reproduction number, *R_0 _*, will be affected by the structure of the networks, investigating network structure alone is not enough to fully investigate the effect of *R_0 _*. To do this, one would need to understand the effect of the individual transmission rates on the probability of a large outbreak.

When comparing the results for small epidemics against those for large epidemics, two factors that differ significantly between the two categories are worth noting: the effect of the probability of transmission via slaughterhouse movements and seed premises size. Large epidemics are up to 28 times more likely for higher levels of slaughterhouse transmission (compared to zero), implying that the characteristics of the network of slaughterhouse links are maintained even when a time component and control measures are added, resulting in connectivity between a higher proportion of premises via this route than via any other route. This result confirms that slaughterhouses are an important factor in this model. The size of seed premises plays a role here as there is an increase in frequency of catching team and slaughterhouse visits to larger premises (Additional File 1 Figure S10). This results in large outbreaks being more likely to occur, as a result of infection in a large seed premises. It is reiterated however that this does not imply that infection seeded in large premises will always result in a large outbreak. Nevertheless, this result does suggests that if premises are to be prioritised during contact tracing, there will be some benefit to targeting large premises ahead of smaller ones in a epidemic situation. Further investigation into all premises included in these epidemics to identify whether the same premises are included in the large epidemics is highlighted here as an area for further research. This will also identify premises that might be considered particularly high risk.

We note that all slaughterhouses that appear in the movement data analysed are recorded as slaughtering birds from farms that are not visited by the catching company studied. This implies that the network of premises studied is not closed; with up to 131 additional farms sending birds to the same slaughterhouse (unpublished data), the possibility of disease spreading into other sub-networks within the industry is potentially high. It is therefore very important to ensure the data held on slaughterhouses and their customers is both complete and up to date. This will enable better prioritisation of the potentially large number of premises that could undergo surveillance in an outbreak situation.

Our results show that the distribution of poultry premises in GB is not dense enough for airborne transmission of AIV contribute significantly to between premises spread amongst premises recorded in the GBPR, so long as the distance for airborne transmission is less than 500 m. This has not been the case in past outbreaks in other countries, such as the Netherlands and Italy, where local spread is likely to have played a role in the transmission of disease from one farm to another. Should a virus strain that can easily transmit via airborne transmission be modelled, then local spread may result in spread between premises that have no other direct connections. For other virus strains, this could have a large impact on the proportion of outbreaks resulting in spread beyond the seed premises and the maximum epidemic size. This implies that there is possible scope to reduce the size of the 10 km SZs, freeing resources for use elsewhere. This could be explored further by using network data currently available to explore how large a SZ should be, taking into account resource constraints and simulating over a range of assumptions regarding transmission rates. The mean number of premises affected by an epidemic may be dependent not only on the underlying epidemiological parameters, but also on the total resources available. Resource constraints were not included in this model but the model could be adapted to aid future work in this area, important for exploring optimal resource allocation in order to provide the most efficient detection of AIV and the curtailing of the outbreak.

## Conclusions

Previous work has shown that large proportions of the poultry industry are potentially connected by catching companies and by slaughterhouse [10]. However, such analyses did not take into account the restriction in the number of interaction events that could occur over the course of a typical infectious period. Including these effects, such as via the explicit spatio-temporal simulations explored in this study, shows that such restrictions can have an important role in determining disease spread risk.

In line with previous work [10], we have shown that slaughterhouses connect the highest number of premises in the poultry industry. Furthermore, the potentially high frequency of company personnel between farms renders this type of movement more important in the beginning of an outbreak. Contrary to expectations, however, the frequency of movements of catching teams between premises is not high enough to connect large numbers of premises, reducing the potential for a large outbreak spread via this route, in GB. The size of seed premises played a role in final epidemic size suggesting that there will be some benefit to targeting large premises ahead of smaller ones in an epidemic situation. The ability of the virus to jump from one sector of the industry to another highlights the importance of keeping data on movements on and off poultry farms both detailed and up to date.

## Authors' contributions

MA designed the study. JED collected the data. JED, RRK and IZK analysed the data. JED wrote the simulation model. JED wrote the manuscript with input from MA, RRK and IZK. All authors have read and approved the final manuscript.

## Supplementary Material

Additional file 1 **Descriptive analysis of catching company data**. Click here for file

Additional file 2 **Simulation modelling methods and outputs**. Click here for file

Additional file 3 **Sensitivity to spatial spread**. Click here for file

Additional file 4 **Results - supplementary tables**. Click here for file

Additional file 5 **Maximum distance between infected premises**. Click here for file
