# Supplementary material for: The potential spread of highly pathogenic avian influenza virus via dynamic contacts between poultry premises in Great Britain
Source: BMC Vet Res. 2011 Oct 13;7:59. doi: 10.1186/1746-6148-7-59 (PMC3224601; doi:10.1186/1746-6148-7-59)
Supplement: Additional file 1 — Descriptive analysis of catching company data. [file 1746-6148-7-59-S1.PDF]

S1 Additional File 1 - Descriptive analyis of catching company data.

S1.1 Individual network characteristics

For these data, a connection was assumed between premises if the two premises were visited on the same day. Under this assumption, over the time period of 936 days, catching teams connected 317 of the 415 (76%) poultry premises visited. The remaining premises were visited either as a one off, or were never visited on the same day as other premises, by the same catching team. Using Tarjan's algortithm [Sedgewick, 2001] to identify clusters of premises that are connected by catching teams, a total of 12 disjoint clusters were found, 11 of these 12 clusters contain fewer than 5 poultry premises. This implies that, over the time period studied, almost 300 premises are connected in at least one direction. In fact, each poultry premises was connected, by catching teams, to an average of 3.98 other premises over the time period studied (this figure, which describes the mean degree per poultry premises, excludes self-loops and counts repeated links only once).

Over the same time period, slaughterhouses connected 391 of the 415 (94 %) poultry premises, immediately suggesting that this network is better-connected than that of premises linked by catching team. These data contained 4 clusters (excluding isolated nodes), one containing 383 premises and the remaining three with four or fewer premises. Each premises was connected, via slaughterhouses, to an average of 15.33 other premises during the time period studied.

The in- and out-degree distributions for premises linked by catching company are given in Additional File 1 Figure S1. In order to show that the network of premises connected by catching team is approximately scale free (i.e. is made up of a small number of highly connected nodes and high number of nodes with a small number of connections), a power-law distribution was fitted to both in- and out-degree to give the number of nodes, x_{in} and x_{out}, with degree, d, as shown in Equation (1) (corresponding R^2 values for in- and out-degree distributions were $R^2_{in} = 0.92$ and $R^2_{out} = 0.86$).

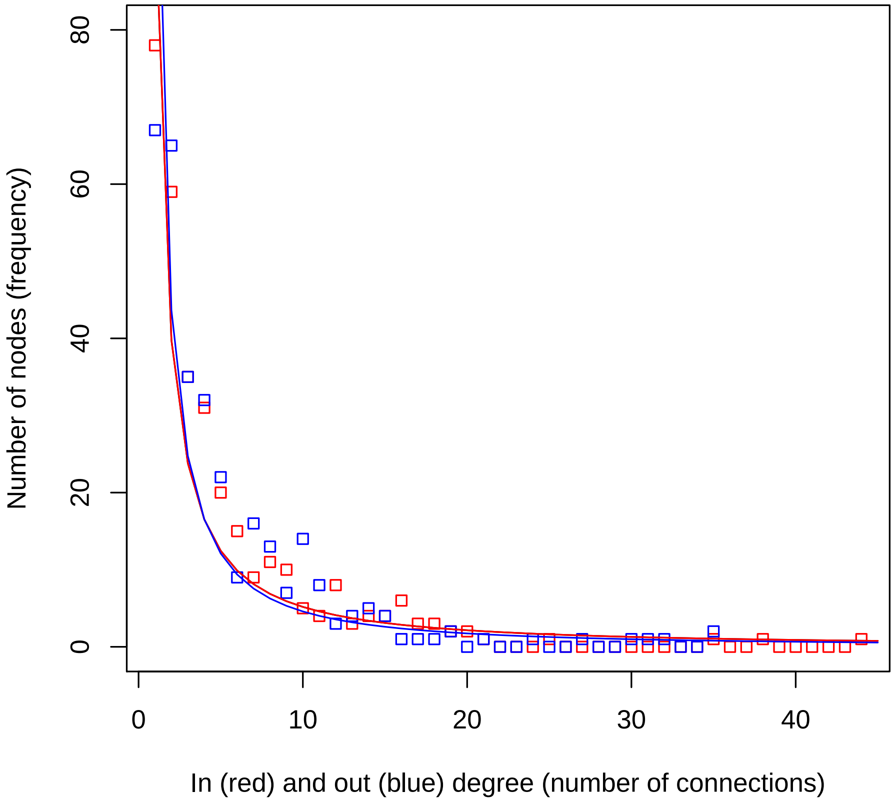

Figure S1. Degree distribution for premises linked by catching company. In-degree = red, out-degree = blue. Points represent true data, lines represent fitted power-law.

$$x_{in} = 95.73x^{-1.27}, x_{out} = 115.20x^{-1.4} \quad (1)$$

For slaughterhouse linked movements, the in- and out-degree distributions, shown in Additional File 1 Figure S2, could not be characterised by a power-law distribution (the R^2 values for a fitted power-law distribution were 0.49 and 0.43 for in- and out-degree, respectively). The slaughterhouse degree distribution showed a distribution that is closer to a Poisson distribution. However, as the mean and variance are not equal, a Poisson distribution could also not be fitted to the data. Despite displaying Poisson characteristics, implying the network is random, the

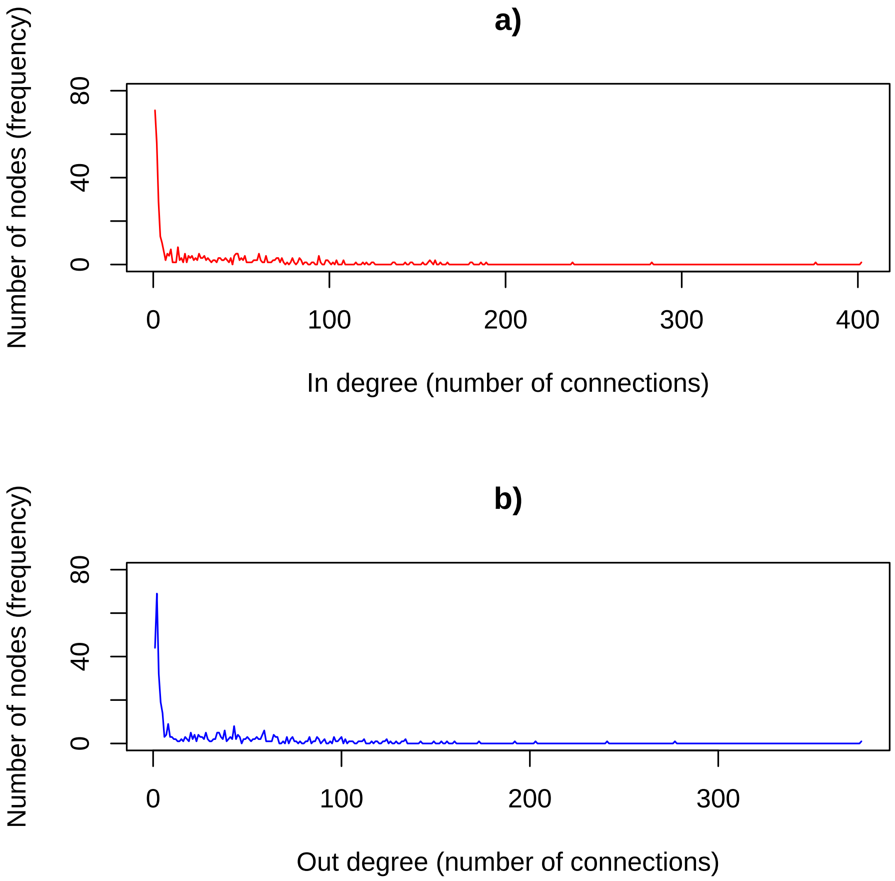

Figure S2. Degree distribution for premises linked by slaughterhouse. (a) In-degree (red) and (b) out (blue).

data showed some exponential decay, which implies that the network may be held together by only few 'hub' nodes, with high degree.

S1.2 Frequency of movements per day

If all links between premises associated with the Catching Company are considered at once (as previously assumed [Dent et al., 2008]) and a per link probability of transmission assumed between farms that are linked, then links between the same premises on different days become important as they can increase the probability of disease transmission between premises. However, in reality AIV is not likely to transmit over such an extended time period and so

the frequency of movements on a daily basis may be more important to consider than that of connectivity over a longer time period.

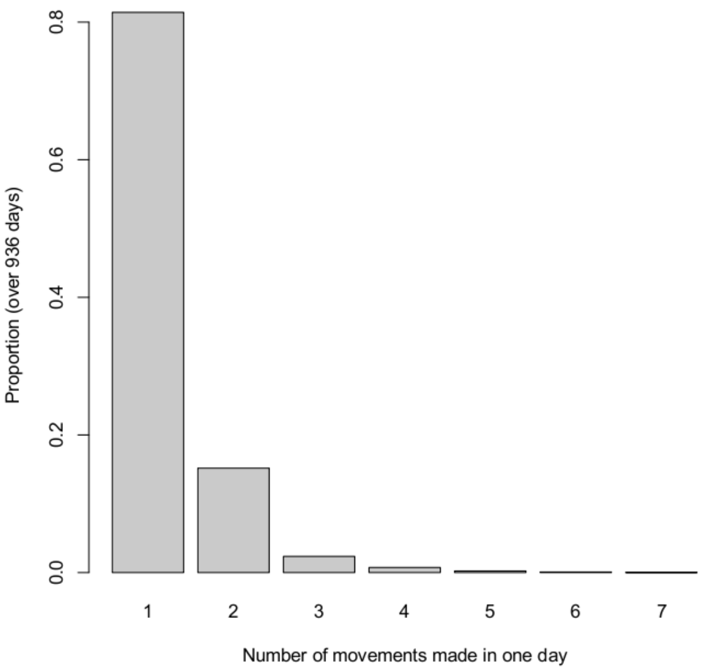

Figure S3. Number of poultry premises visited per catching team per day.

The average number of connections per node for premises connected on the same day is 0.19 for connections made by catching team movements and 2.53 for slaughterhouse linked movements (this figure assumes that self-loops -where the same premises is visited multiple times on one day by the same catching team or slaughterhouse vehicle- are not accounted for). There were a large number of visits to premises that did not result in onward movements. When an onward movement did occur, a mean of 1.22 (variance = 0.39) and 3.33 (variance = 9.5) premises were connected by catching team and slaughterhouse, respectively. Additional File 1 Figures S3 and S4 show the empirical distributions of data describing how many premises are visited per day by catching teams and slaughterhouse vehicles, over the full 936-day period.

Additional File 1 Figure S3 shows that in approximately 84% of cases, only one premises is

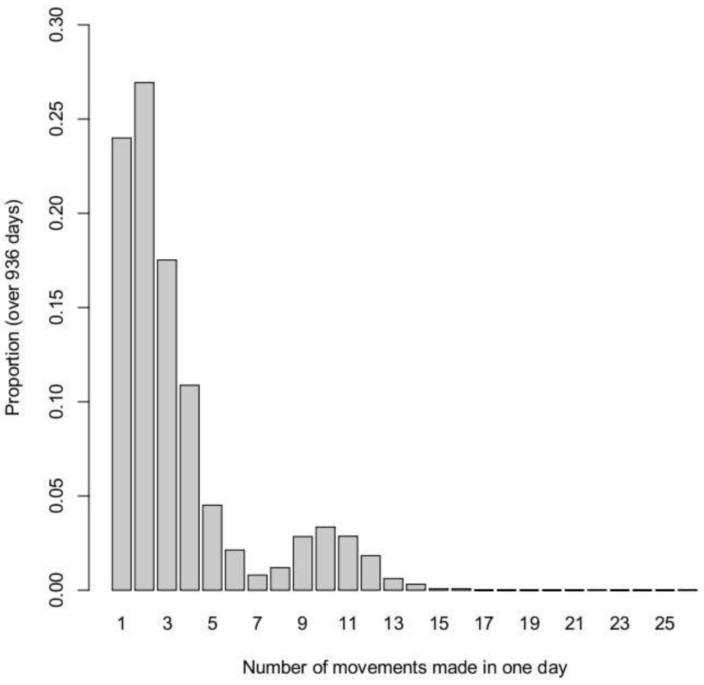

Figure S4. Number of poultry premises visited by slaughterhouse vehicles, per day.

visited by a catching team on a given day, implying that the event that catching teams visit more than one premises in a day is rare.

Despite the low probability that more than one premises was visited in a day by catching teams, the data show that up to seven premises were visited in one 24-hour period (see also Additional File 1 Figure S3), so it is not wise, at this stage, to eliminate this as an important transmission route between premises. In addition, the data show that only 24% of poultry premises are serviced by a single catching team, with some premises being visited by up to 30 different catching teams (Additional File 1 Figure S5) within the time period studied. Although no premises were visited on the same day by different teams, this results implies that there is mixing between teams and that catching teams can indeed connect multiple farms.

Conversely, for slaughterhouses, only 24% of visits were to single premises, implying that the event that a slaughterhouse vehicle visited more than one premises on a single day was much

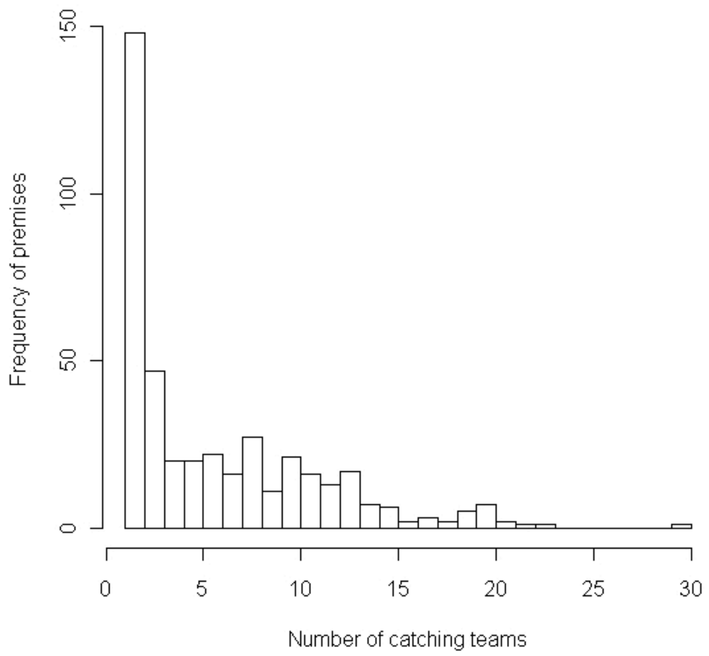

Figure S5. Number of catching company teams associated with poultry premises.

less rare. This occurs due to the lower number of slaughterhouses (compared to catching teams) associated with the Catching Company. Furthermore, when more than one poultry premises was visited by a slaughterhouse vehicle, Additional File 1 Figure S4 shows that up to 26 movements (to different premises) were made in one day. This implies that, even when frequency of movements are taken into account, slaughterhouse-related movements might be frequent enough to cause an outbreak to reach multiple premises. Interestingly, Additional File 1 Figure S4 also shows that there is a bi-modal pattern in these data. There is a large peak at 2 movements per day and another smaller peak at 10 movements per day. This could be related to the capacity of the slaughterhouses to handle birds. An explanation for this could be that it is possible that larger slaughterhouses have the capacity to visit an average of 9 - 12 farms per day, whereas the smaller ones (of which there may be more) can visit only two or three premises per day. Visitng over 13 or 14 farms a day appears to be only occur in exceptional

cases. Investigating this further could provide an area of further research.

Consistent with the data in the PND, although just over half of premises send birds to just one slaughterhouse, some premises send birds to multiple slaughterhouses (up to six according to data from the Catching Company and up to eight according to the PND). This may be a result of slaughterhouses operating on a species-specific basis, so farms housing multiple species send birds to multiple slaughterhouses. According to the movement data from the Catching Company, premises using the Catching Company send birds to one or more of eight slaughterhouses. Data collected from 96 slaughterhouses however, suggests that up to 35 slaughterhouses are associated with the premises that use the Catching Company. This suggests that either premises use multiple catching companies, or premises catch birds themselves and send to multiple slaughterhouses. Due to the ability of slaughterhouses to connect a larger number of premises than catching companies, it is important to determine which case is most likely. Results from the static network analyses presented in [Dent et al., 2008] suggest that it is more likely that premises use multiple slaughterhouses than they do multiple catching companies.

S1.3 Movement dependent on farm size

There is evidence to suggest that the probability that more than one farm is visited by a catching team, in a day, is related to the size of the first farm visited.

Figures S6 and S7 show the empirical distributions of data describing whether or not an onward movement was made (by catching teams) for different farms sizes (measured by the number of houses (S6) and the number of birds (S7) on a farm).

The light grey peak in Additional File 1 Figure S6 shows that onward movements were most likely to have occured after a medium sized farm had been visited (farms with 10-11 houses). We would expect these farms to be operating in cycles, so that there are always birds on the farms and only a small number of houses are visited per catching team visit. The dark grey peak at farms of size 12-14 houses suggests that no onward movement was most likely to have occured after large farms had been visited (however, there is only a slight difference in the

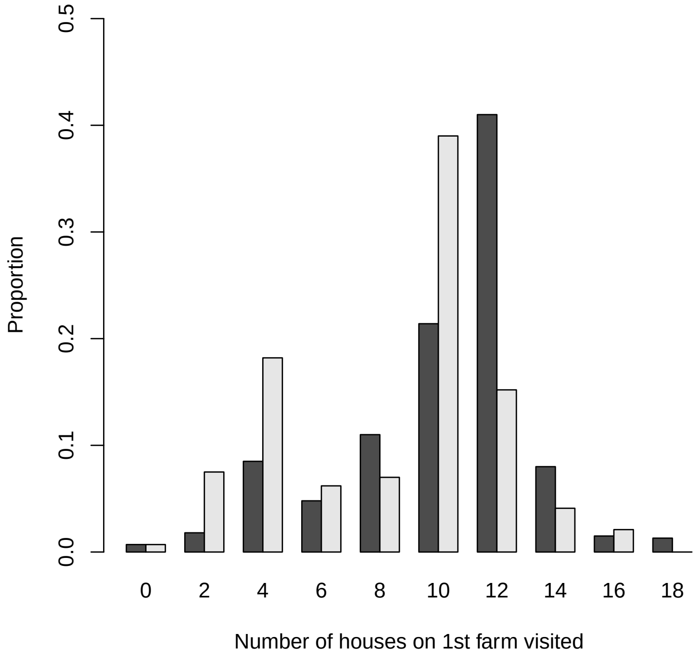

Figure S6. Distribution of whether or not onward movements (dark grey, no onward movement and light grey, onward movement) were made by a catching team, given the number of houses on the first farm visited.

location of the light and dark grey peaks). These farms may be operating an all-in-all-out procedure, where all birds are caught at once and sent to slaughter. There was another small peak of onward movement from small farms (with 4-5 houses), implying that catching team visits to small farms are likely to have resulted in the team visiting other premises on the same day. When number of birds was used to determine farm size (Additional File 1 Figure S7), then onward movement was still most likely to occur from medium to large farms (housing between 240,000 and 280,000) birds and no onward movement most likely from larger farms (housing 320,000 to 360,000 birds). The small-farm peak seen in Additional File 1 Figure S6 can also be seen for onward movements from farms housing 40,000 to 80,000 birds. These results imply that when catching teams visit more than one farm, they are most likely to have come from a small to medium sized farm first. However, if they visit only one farm in a day, it is most likely

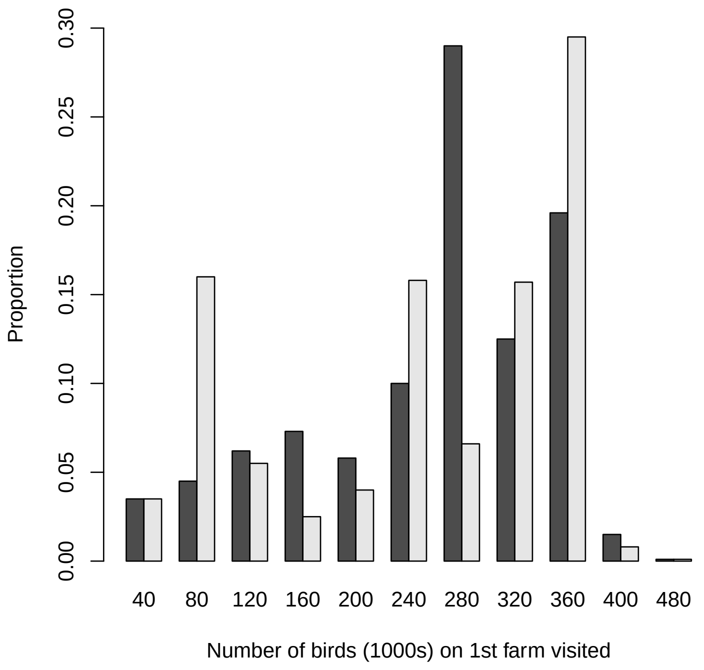

Figure S7. Distribution of whether or not onward movements (dark grey, no onward movement and light grey, onward movement) were made by a catching team, given the number of birds (in 1000s) on the first farm visited.

that this farm is a large farm.

Similar conclusions however, cannot be drawn from the empirical distributions of data describing whether or not an onward movement was made by slaughterhouse vehicles and personnel. Additional File 1 Figures S8 and S9 show the distribution of how often onward movements occurred according to farm size (houses and number of birds), for movements made by slaughterhouse vehicles and personnel. Additional File 1 Figure S8 shows that onward movements were least likely to have occurred after visiting large farms (more than 7 houses) and most likely to have occurred when the first farm visited has four or fewer houses. There is another peak at farms with 7 houses, which occurs because there are two farms with 7 houses that are frequently visited by vehicles from the same slaughterhouse. Although no onward movements were most likely to have occurred when a farm with four houses was visited by a slaughterhouse

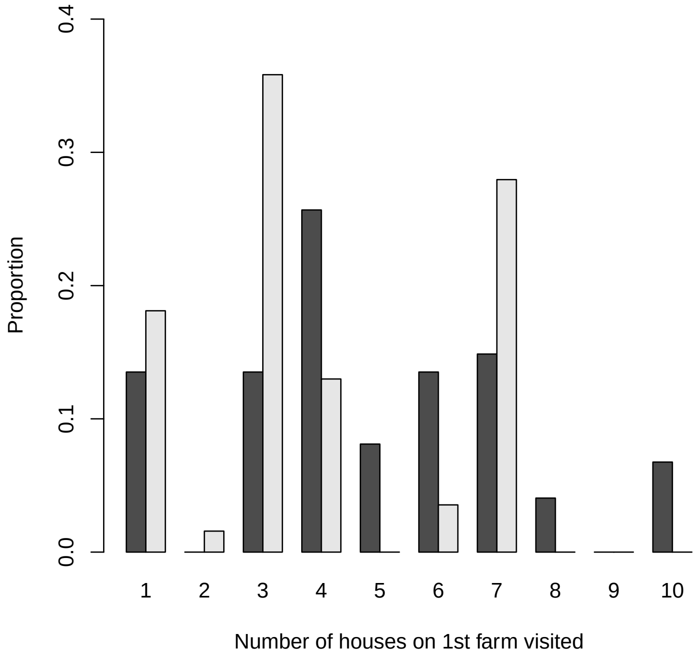

Figure S8. Distribution of whether or not onward movements (dark grey, no onward movement and light grey, onward movement) were made by a slaughterhouse vehicles, given the number of houses on the first farm visited.

vehicle (dark grey peak in Additional File 1 Figure S8), patterns in the data are not evident. When the distribution was replotted against the number of birds on a farm (Additional File 1 Figure S9), the data tell a different story. First of all, the data show that the first farm to be visited by a slaughterhouse vehicle was almost always a small to medium farm (fewer than 200,000 birds). For this reason, if only one farm is visited is is likely to be small, if more than one farm is visited, it is likely that a small farm was visited first. The data for farm size by number of birds supports that of farm size by number of houses in that onward movements did not occur in large farms. This could suggest that slaughterhouse vehicles require a long time to load and transport birds from large farms, leaving no time (and perhaps no free equipment) in the day for movement to other farms. Interestingly, no onward movement was most likely to occur from farms housing 40,000 or fewer birds. This implies that all birds on these farms are

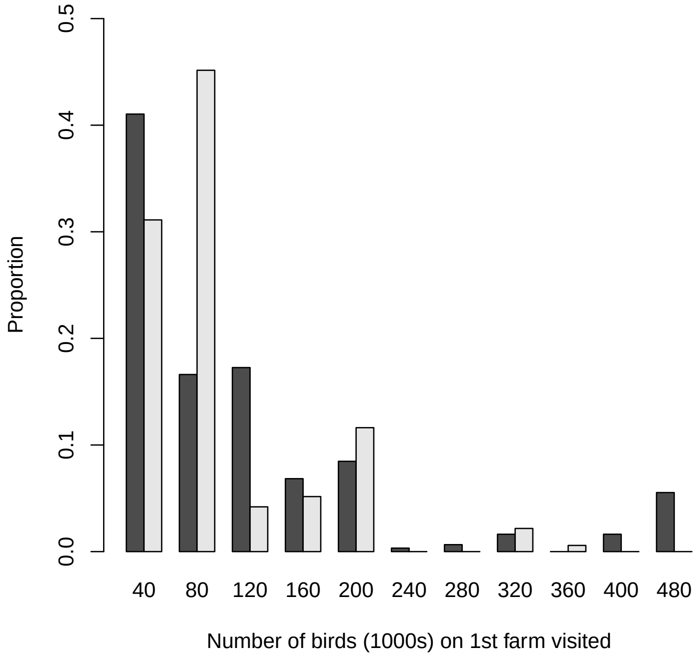

Figure S9. Distribution of whether or not onward movements (dark grey, no onward movement and light grey, onward movement) were made by slaughterhouse vehicles, given the number of birds (in 1000s) on the first farm visited.

taken to slaughter in one batch (i.e. they operate on an all-in-all-out basis and not in cycles). For slightly larger farms (40,000 to 80,000 birds), onward movements were more likely to have occurred, perhaps because these farms operate in cycles, so fewer birds are taken to slaughter in one batch, allowing vehicles to collect birds from other farms in order to fill the truck. In conclusion, the patterns of onward movements of slaughterhouse vehicles cannot easily be predicted from farm size, though the data do suggest that there is some prioritisation of the order of visits, in that small farms are more likely than large farms to be visited at the beginning of a day.

S1.4 Repeated movements

Under the assumption that risk of infection is related to the frequency of visits, large premises are at a higher risk of becoming infected via the movement of catching teams (and slaughter-house vehicles).

According to the data, 51% of links between premises that are created by the movement of catching teams were repeated at least once over the time period studied. Seventeen percent of premises were only visited once in the data set. Approximately 1% of premises were visited over 200 times, with one premises being visited 370 times (one visit every 2 to 3 days). According to the GBPR, this premises consists of seven houses of 36,000 broiler chicks per house, so if the cycle in each house is one week apart and each house is visited separately, thinned (once birds reach a certain size, a small proportion are removed, freeing up space for remaining birds to grow bigger) and has part depopulation (not all houses are emptied of birds at the same time, so that there are always birds present on the farm, and depopulation takes place over a prolonged period of time), at different times, it is possible that the site is visited every few days. Generally speaking, larger premises are visited more frequently than smaller premises.

Additional File 1 Figure S10 shows the distribution of the number of visits made by catching teams, according to premises size. The Figure shows that large premises (>200,000 birds or more than 10 houses) were visited more frequently, perhaps as a result of 'thinning' (over 100 visits in the time period (936 days), corresponding to visits made every 10 days or more frequently). Interestingly, small and medium premises are less frequently visited (the majority receiving fewer than 100 visits over the 936 day period). There could be several explanations for this: such premises may be using multiple catching companies (or catching birds themselves) or they may operate an all-in-all-out procedure as there are not enough birds on the farm to justify a stratified production procedure. It is noted that other factors, such as biosecurity measures employed by farm staff for example, may vary according to farm size. This is less relevant here as the biosecurity measures employed by personnel associated with catching company are set by the catching company rather than by the farm being visited.

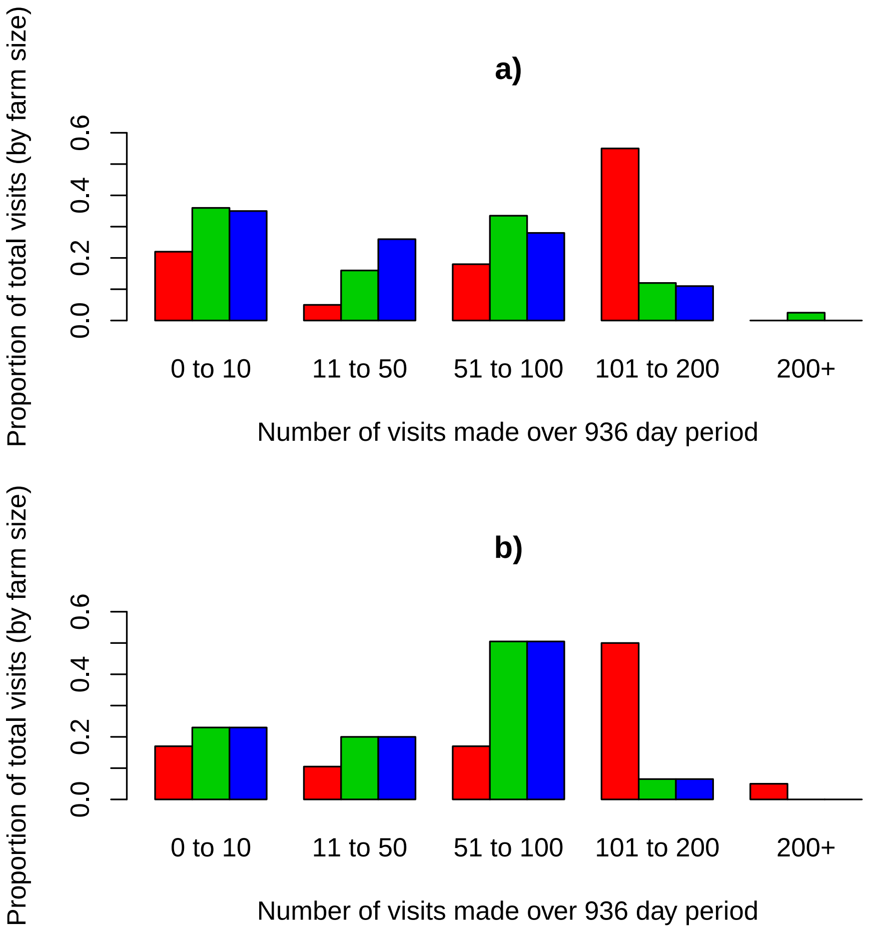

Figure S10. Distribution of number of catching team visits per premises for (a) premises size defined by number of houses (red = 10 or more houses, green = 5 to 10 houses, blue = 0 - 5 houses) and (b) premises size defined by number of birds (red = more than 200,000 birds, green = 100,000 - 200,000 birds, blue = 0 - 100,000 birds).

S1.5 Distance between associated premises

In an outbreak situation, surveillance and protection zones are set up at 10km and 3km, respectively, around infected premises. If these zones are to be effective in controlling disease, then movements between premises should be restricted to occurring within these zones. For these data, the majority of premises are situated more than 3km (the current PZ put around infected premises) from each other.

Additional File 1 Figure S11 shows the distribution of the number of premises located within

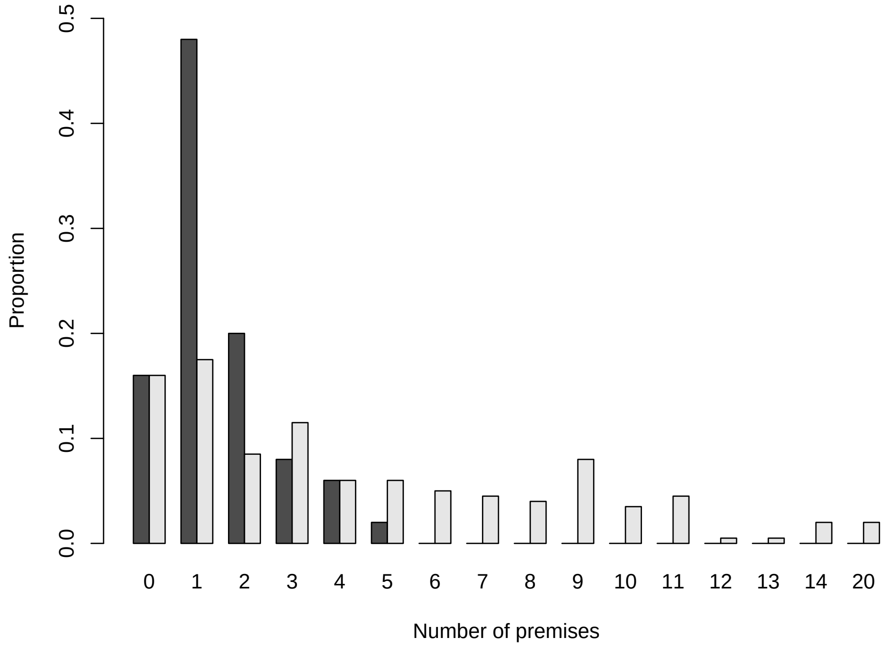

Figure S11. Distribution of the number of premises located within 3km (dark grey) and 10km (light grey) of each premises associated with the catching company.

3km (dark grey) and 10km (light grey) of each premises associated with the Catching Company. The figure shows that almost 50% of premises have only one other premises located within 3km, with no more than 5 premises located within 3km of each other. The figure also shows that there are up to 20 premises (corresponding to approximately 5% of premises) located within 10km of each other. As only a maximum of 5% of premises are located within 10km of each other, this implies that there may be many premises that are associated with the Catching Company that would not be located within the surveillance zone of a (potentially) infected poultry farm. In fact, for these data, approximately 16% of premises are located more than 10km away from all other premises. However, when GBPR premises are added to the data, a total of 1987 additional premises are located within 3km of the studied premises and 9298 premises within 10km. We further note that a total of 504 GBPR premises are located within 500m of a premises associated with the catching company, suggesting that a limit of 500m for

spatial spread (that is assumed in the simulation model) could still result in transmission via this route.

When we consider the distance between premises that are linked by catching teams and the distances travelled between premises and slaughterhouses, we see that the majority of linked premises are further than 10km apart.

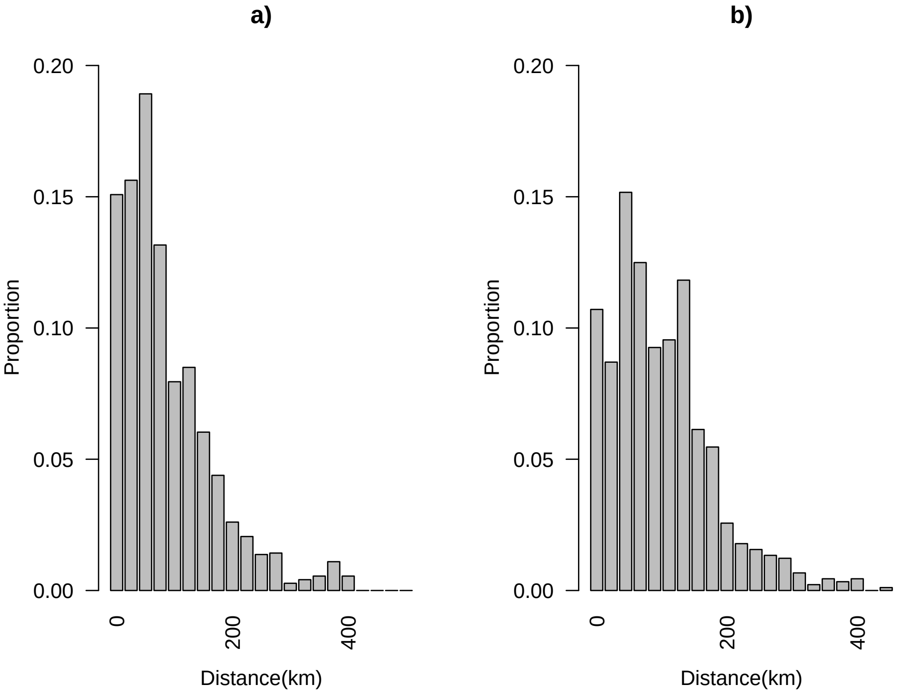

Figure S12. Distribution of distances travelled for (a) catching teams between premises and (b) from premises to slaughterhouse.

The movements of catching teams between premises and the movements made from premises to slaughterhouse could cover long distances (Additional File 1 Figure S12), resulting in the potential for geographically widespread dissemination of virus. Only 28% of catching team movements were made between premises less than 10km apart (within the current SZ), with some catching teams travelling very long distances between premises on the same day. The increase in the proportion of movements that are greater 300km apart is caused by catching teams visiting a single premises located in a more remote area of GB. The Euclidean distance

travelled by slaughterhouse vehicles, from premises to slaughterhouse was also relatively long, with a mean distance of 106.2km (with a large standard deviation of 73.15, implying diversity in the data).

When road distance is considered as a measure for distance, there is little difference in the results obtained.

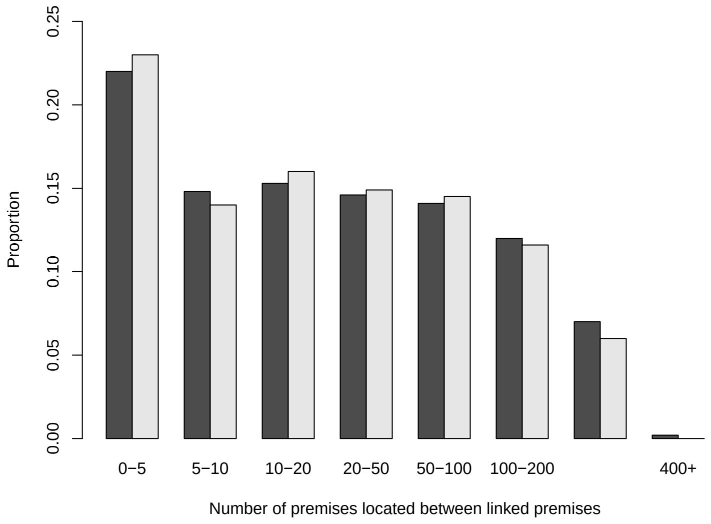

Figure S13. Comparison of road and Euclidean distances. Distribution of the number of premises located between premises that are linked, according to Euclidean distance (dark grey) and road distance (light grey).

Additional File 1 Figure S13 shows the distribution of the number of premises located between two linked premises, counted using Euclidean distance and road distance to measure the distance between linked premises. The figure shows that there is little difference between using road and Euclidean distance to measure distance between linked premises, for these data. Road density is high in the South and East of GB, compared to the North and West and, given that a high proportion of premises associated with the Catching Company are located in the East of GB, this might explain why there is little difference between road and Euclidean distance for these data. Furthermore, the figure shows that most movements are made between premises that are close to one another, with over 20% of links occurring from one premises to one of the five

closest neighbouring premises. These results thus suggest that the use of Euclidean distance as a distance measure for these data is acceptable.

S1.6 Owner movements

In addition to these data, it was assumed that links could occur because of premises being linked by poultry company i.e. poultry premises with the same owner. Owner movement data were not available for analysis and were therefore simulated, based on expert opinion. However, information about which poultry premises belong to which company was obtained from a combination of the Catching Company data and the PND. Similar data were not available for farms that were added to the dataset from the GBPR. This is highlighted as an area for further study.

Of the 415 farms in the data set, 114 were associated with a multi-site poultry company. There were 10 multi-site companies associated with the Catching Company, the largest of which contains 43 of the poultry premises associated with the Catching Company. The number of links per node via owner (representing the mean degree) for these data is 24 premises. The owner network clearly represents only a small proportion of the 415 farms that use the Catching Company and so we might expect that transmission via this route is not likely to be the most influential to the model results. It is also noted that there is no overlap between companies, so each poultry farm can only be associated with one poultry company.

Bibliography

[Dent et al., 2008] Dent, J., Kao, R., Kiss, I., Hyder, K., and Arnold, M. (2008). Contact structures in the poultry industry in Great Britain: Exploring transmission routes for a potential avian influenza virus epidemic. *BMC Veterinary Research*, 4(1):27.

[Sedgewick, 2001] Sedgewick, R. (2001). *Algorithms in C, Part 5: graph algorithms*. Addison-Wesley Professional.
