# Supplementary material for: The potential spread of highly pathogenic avian influenza virus via dynamic contacts between poultry premises in Great Britain
Source: BMC Vet Res. 2011 Oct 13;7:59. doi: 10.1186/1746-6148-7-59 (PMC3224601; doi:10.1186/1746-6148-7-59)
Supplement: Additional file 2 — Simulation modelling methods and outputs. [file 1746-6148-7-59-S2.PDF]

S2 Additional File 2 - Simulation modelling: methods.

The simulation model, written in C language, is designed to simulate the spread of avian influenza virus between a sample of premises in Great Britain (GB). Virus is spread via the movements of catching teams, slaughterhouse vehicles or personnel, company personnel or by limited airborne spread.

The simulation can be broken down into a number of steps as shown in Additional File 2 Figure S1, summarised by Additional File 2 Algorithms S1 to S5.

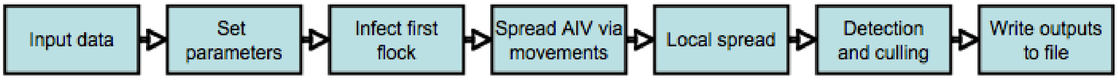

Figure S1. Programme design for AIV simulation.

S2.1 Simulation model: input data

Input data are read in to the programme as a series of text files as described in Additional File 2 Table S1. Movement data of catching teams (CC) and slaughterhouse vehicles (SH) were obtained for a period of 936 days, for a sample of 415 farms in GB. These farms will be referred to as 'network' farms. Movements of farm personnel between premises and local spread are simulated based on expert opinion. Data for poultry farms located within 15km of network farms are collected from the GB poultry register and included in the simulation in order to allow for virus to spread outside the network of farms for which we have movement data.

Table S1. Input data for AIV simulation (GB)

**File name**	**Size (cols x rows)**	**Description**
Link_index	Number link types x Number farms in network*	Number of farms connected to each farm by each link type.
Continued on next page		

Table S1 – continued from previous page

File name	Size (cols x rows)	Description
GBPRlocal15	5 x Number farms in study region (within 15km of network farms)	Farm input data: farm number, farm ID (from Defra database - not required), easting, northing, size (birds).
GBPR_local15_flocks	6 x Number flocks** in region studied	Flock input data: farm number, flock number, easting, northing, size (birds), species type.
Nwork_SZ10	19 x Number Farms in network	Vector of network farms within 10km of each network farm.
Nwork_SZ10index	1 x Number Farms in network	Number of network farms within 10km of each network farm.
flockprem_GBPR_Nwork	1 x Number flocks	Which farm each flock is in.
flocksinprem_GBPR_Nwork	1 x Number farms	Number flocks on each farm.
Premtoflock_GBPR_Nwork	23 x Number Farms	Vector of flocks that are on each farm.
own_links	2 x Number of links	One link per row: Farm A, Farm B.
Events_CC	7 x Number catching company events	List of farms visited by each catching team per day. One team per day per row.
Width_CC	1 x Number catching company events	Number farms visited per day per team. Row number corresponds to row in Events_CC.
Date_CC	1 x Number catching company events	Day of catching team event. Day 1 = starting date. Row number corresponds to row in Events_CC.

Continued on next page

Table S1 – continued from previous page

File name	Size (cols x rows)	Description
Time_CC	4 x Number days	Day number; number CC events for that day; 1st CC event, last CC event. CC event = row number from Events_CC.
Events_SH	7 x Number catching company events	List of farms visited by each catching team per day. One team per day per row.
Width_SH	1 x Number catching company events	Number farms visited per day per team. Row number corresponds to row in Events_CC.
Date_SH	1 x Number catching company events	Day of catching team event. Day 1 = starting date. Row number corresponds to row in Events_CC.
Time_SH	4 x Number days	Day number; number CC events for that day; 1st CC event, last CC event. CC event = row number from Events_CC.
Non_Nwork_SZ10	5 x Number non-network farms within 10km of network farms	Row ID, Farm number, farm ID, easting, northing.

*Network farms refer to farms in the study region for which movement data are available, or simulated. **Flocks: we assume that all birds of the same species and purpose (eg layer chickens or meat chickens) are kept together on a farm and we therefore refer to them as a single flock, so a farm that houses ducks, broiler chickens and layer chickens will be recorded as a farm of three flocks.

The movement data are then transformed into an array with dimensions as shown below. The array describes the exact order of events per flock per day, these data are combined with

information about the potential links that may occur due to premises belonging to the same integrated company and the distance between the premises. Each entry in $array[0]$, $array[1]$ and $array[2]$ is a flock ID number, the entries in $array[3]$ are distances (m) corresponding to links in $array[2]$:

$array[0][][]$ =(Number network premises)∗(Number days in data)∗(number CC visits per premises per day)

$array[1][][]$ =(Number network premises)∗(Number days in data)∗(number SH visits per premises per day)

$array[2][]$ =(Number network premises)∗(Number premises in same integrated company)

$array[3][]$ =(Number network premises)∗(Distance between premises linked by integrated company)

S2.2 Simulation model: set parameters

Parameters are set in the programme as shown in Additional File 2 Table S2.

Table S2. Parameters (inc. matrices) used in simulation model

**Parameter**	**Description/comments**
Number iterations	100
Run time	Number of days to run model for = 50 days
Random number	Pseudo random number generator, using real time as seed
Random farm	Farm chosen at random, on which to seed infection
Random time	Day, chosen at random, on which to seed infection
Max Dist	Maximum distance between infected farms
Species type	Type of species in flock
Species prob	Probability of a given species being visited varies according to species, based on the number of each flock type visited, where data were available chicken = 0.71, ducks/geese = 0.16, Turkeys = 0.12, other = 0.01 (2dp).
Continued on next page	

Table S2 – continued from previous page

Parameter	Description/comments
Probability of transmission via local (airborne) transmission given by distance kernel	Probability of transmission between two farms within a certain distance of each other is based on a distance kernel described by Boender (2007) in analysis of geographical spread of infectious diseases (see section A-1.5, equation 1). Maximum transmission set to 0.01 at zero meters (ie. between flocks on same farm). Maximum distance over which airborne spread can occur = 500m.
Probability of transmission (via farm personnel, catching team, slaughterhouse vehicle)	Transmission rates between premises are currently unknown so they are varied between 0 and 0.2 in a stepwise fashion, with an extra parameter value added at 0.001. This results in 22 parameter values per potential transmission route.
Time to detection	Number of days between infection and detection (at farm level). It is assumed that detection occurs within 6 days of infection, with a 2-day latent period, described by a triangular distribution based on mean time to detection of 4 days to estimate time to detection for premises outside a PZ/SZ and mean time to detection of 3 days inside these zones. Time to detection is the same for all species apart from ducks, where we add 15 days on the estimated time to detection.
Time to culling	3 days after detection outside PZ/SZ, 2 days after detection in SZ and 1 day after detection in PZ. Based on Defra report from outbreak in GB 2007.
Probability of staff working on multiple farms	Expert opinion. Dependent on farm size and species as described previously. We assume small premises are more likely to share staff. Assume premises only share staff within 35km radius.
Probability of area manager visit	Expert opinion (discussed below). Fewer visits expected to layer farms.
Probability of vet visit	Expert opinion (discussed below).
Infected Farm List	List of premises infected during outbreak
Infection Time	Day premises becomes infected
Detection Time	Day infected premises are detected
Culling Dates	Day infected premises are culled
Farm Inf	Infectious state of a premises (susceptible (0) infected (1), detected (2), culled (-1))
Proportion positive	Proportion outbreaks that result in spread beyond the seed premises, for each parameter set.

Continued on next page

Table S2 – continued from previous page

Parameter	Description/comments
SZ Matrix	Premises in surveillance and protection zones

S2.3 Simulation model: infect 1st flock

For 100 simulations of each combination of transmission parameters, the random number generator chooses a premises in the network to infect and a time to infect the premises from day zero to day 886 (total number of days minus number of days epidemic is left to run for). If the premises is visited within 15 days of infection, then the programme chooses a flock on the seed premises to infect, based on the species type, where known, such that chickens are the most likely to be infected (as they are the most likely to be visited by the catching company). If the premises is visited within 15 days of infection, transmission can occur via movement of catching teams and slaughterhouse vehicles. Otherwise, transmission is restricted to local spread or spread via company personnel movements (see Additional File 2 Algorithm S1). The infectious state of the first premises is set to 1 and the model enters the 'transmit disease' stage, where it first transmits infection via movements and then via local spread.

Algorithm S1: AIV MAIN FUNCTION(*pseudocode*)

```
for transmisison probabilities (CC, SH, own) ← 0 to 0.2
  do {
    for iterations ← 1 to 100
      do {
        choose a random farm to visit
        choose a random time to start infection
        for time ← t to t+50
          do if farm visited within 15 days of start time
            then {
              choose a flock to infect based on species type
              set detection dates for 1st infected farm
              set culling dates for 1st infected farm
               Transmit AIV as in Alg. A-5
            }
        Transmit AIV via local spread as below

        comment: Update SZ and PZ
        for number farms ← 1 to Number newly infected farms
          do {
            find all farms within 10km and label as in SZ
            find all farms within 3km and label in PZ
          }

        comment: Do detection and culling of infected farms
        for infected farms ← 1 to total number infected farms
          do {
            if Detection Time of Infected Farm = t
              then update status to detected
            if Culling Dates of Infected Farm = t
              then update status to culled
          }
      }
    output data
  }
```

S2.4 Simulation model: spread AIV via movements

Movements of catching teams and slaughterhouse vehicles are determined entirely by the real-time movement data, this means that infection via these transmission routes is independent of farm type and dependent only on the movement between an infected and susceptible premises taking place. Catching team movements always precede slaughterhouse vehicle movements. Spread of infection between premises belonging to the same integrated company, or via spatial transmission are determined stochastically, based on species type and farm size and simulated after infectious movements via catching team and slaughterhouse have occurred. Additional File 2 Algorithm S2 gives pseudocode for the spread of AIV via movements.

Algorithm S2: AIV VIA MOVEMENTS(*pseudocode*)

```
for time(days) ← 1 to 50
  do { for Farms ← 1 to Number of infected farms
       do { for farm visited by CC ← 1 to No. visits in 1 day
              do if farm is already detected/frozen/culled
              then break
              else if infected farm is not yet detected
              then Infect via movements (Alg. A-3 to A-5)
```

S2.5 Catching company and slaughterhouse movements

Once a poultry farm has been infected at random, the programme runs for 50 days (this time was chosen as in test runs of the programme, no epidemic exceeded 50 days under the assumptions made), infecting, detecting and culling premises as follows (shown in Additional File 2 Algorithm S3).

Assuming the seed premises (premises i) is visited by a catching team (and hence a slaughterhouse vehicle) within 15 days of seed infection (day j), then the programme accesses the appropriate place in the links array (see above). For the ith premises, on the jth day, the links array gives a list of all premises that are visited after the ith premises. Given that AIV has an incubation period of only several hours, it is assumed that birds are able to spread disease from the point that they become infected, so that all premises visited after the seed premises becomes infected and on the same day, are susceptible to transmission of disease. Before transmitting disease to susceptible premises, the programme checks that the susceptible premises is in fact susceptible (by checking infectious state of the premises = 0). The programme then checks that the premises does not have any restrictions placed on it (ie is not in a PZ or SZ) and, assuming there are no movement restrictions, proceeds by infecting a flock on the susceptible premises with a probability equal to the probability of transmission via catching company movements (varied between 0 and 0.2). Where the number of flocks on the premises is greater than one, one or more flocks are chosen to be infected according to the probabilities highlighted in Additional File 2 Table S2. A premises is infected when one or more flocks on the premises is infected. If

a premises is visited multiple times in one day, then it is potentially connected to more than one infected premises and the probability of infection is given by Equation (1).

$$P\,(i \text{ gets infected}) = 1 - (\textstyle\prod_j (1 - p_j)),$$
$$\text{for } j \text{ infected premises and } p \text{ probability of infection for each contact.} \tag{1}$$

As soon as a premises is infected, the infectious status of the premises is updated and the premises ID added to the list of infected premises. Detection and culling dates are set (though detection and culling occur at the end of each time step) for each newly infected farm.

This process is then repeated for slaughterhouse-linked movements.

Algorithm S3: TRANSMIT AIV VIA CC/SH(*pseudocode*)

```
for susceptible farm ← 1st farm visited to last farm visited
  do {
    count number of flocks and number of duck flocks
    if susceptible farm has not been frozen
      then {
        choose a flock to be visited by CC
        for susceptible flock ← 1 to number flocks on farm
          do {
            infect using random number generator
            if random num < transmisison probability
              then {
                infect flock
                note change in flock status
                update farm status to infected
                if flock is ducks
                  then make special note
note number of new infections, and date of new infection
comment: set detection time of newly infected farms
comment: if not in PZ or SZ, time to detect is slower
if farm is neither in SZ or PZ
  then {
    use time to detection function: expected = 4days
    if only ducks are infected
      then add 5 days to detection time
    Set culling date to detection date + 3days
  else if farm is in SZ/PZ
  then {
    expected time to detection reduced to 4 days
    if only ducks are infected
      then add 5 days to detection time
    Set culling date to detection date + 2/1 days (SZ/PZ)
```

S2.6 Company personnel movement

The programme then moves on to transmission of disease via company-related movements (referred to as owner movements), as shown in the pseudocode in Additional File 2 Algorithm S4. These movements are simulated in the programme, based on expert opinion from P. Mcmullin (Poultry Health Services, UK). For each infectious premises, the programme determines if the premises is, on each day, sharing staff, according to the probability of staff working on multiple premises as shown in Additional File 2 Table S3

Table S3. Probability of premises share staff, according to premises size (number birds on premises)

Probability of staff working on multiple premises (dependent on farm size)	0.45 (<50,000 birds)<br>0.1 (50,000 to 200,000 birds)<br>0 (>200,000 birds)
Frequency of vet visits	Every 50 days
Frequency of manager visits	Every 10 days (non-layer farms)<br>Every 50 days (layer farms)

If staff shares are found to occur on an infected premises, the programme uses the links array to search for other premises in the same company, within a 35km radius, which can also have a staff share and determines, with the probabilities given in Additional File 2 Table S3, if these premises are also sharing staff on each day of the simulation. If there exists two premises within the same company that are within this region and both 'sharing staff', then we assume that there is a link between the premises and the second premises is classed as susceptible. We also assume that there is a small probability, based on whether or not a premises houses laying hens (Additional File 2 Table S3), that a vet or an area manager will visit an infected premises on each day of the simulation. If this occurs, susceptible farms within the same company are searched for and also visited by the vet or area manager with the same small probability, creating a link between infected and susceptible farms. The programme then infects susceptible farms in a similar way as with catching team and slaughterhouse-related movements; first confirming the infectious status of susceptible premises and then infecting a flock according to species type

and setting detection and culling dates for all newly infected premises.

Once infection has occurred via movements, the programme uses the input file, ‘Nwork_SZ10’ (Additional File 2 Table S1), to create 10km surveillance zones (SZ) and 3km protection zones (PZ) around infected premises (refer to Additional File 2 Algorithm S1) and writes these premises to the ‘SZmatrix’ array (Additional File 2 Table S2). The date at which susceptible premises enter and leave these zones is set so that movement can be frozen within these zones if desired. The programme then adds GBPR premises to the SZ and PZ in preparation for local spread.

Algorithm S4: TRANSMIT AIV VIA OWNER(*pseudocode*)

```
for infected flock ← 1 to total number infected flocks
  do
  { assign staffshare probability, based on farm size
  { determine if layer flock or not
  { assign probability of manager visit
  { for susceptible farms ← 1 to Number farms in company
  {      do { if manager visit occurs on infected and susceptible farms
  {         {      then { assume link between infected and susceptible farms
  {         {           { if random num < transmisison proability for owner
  {         {           {      then { infect a flock according to species
  {         {           {           { note change in flock status
  {         {           {           { update farm status to infected
  {         {           {           { if flock is ducks
  {         {           {           {   then make special note
  {         { repeat for vet visits
  {         { if staff sharing on both farms and distance close enough
  {         {      then { assume link between infected and susceptible farms
  {         {           { if random num < transmisison proability for owner
  {         {           {      then { infect a flock according to species
  {         {           {           { note change in flock status
  {         {           {           { update farm status to infected
  {         {           {           { if flock is ducks
  {         {           {           {   then make special note
  { note number of new infections, and date of new infection
  { set detection time of newly infected farms as in Alg. A-3
  { set culling dates as in Alg. A-3
```

S2.7 Local spread

Irrespective of infectious status, local (airborne) spread can occur between all premises within a predefined distance, as shown in Additional File 2 Algorithm S5.

Algorithm S5: AIV LOCAL TRANSMISSION(*pseudocode*)

for *flocks* ← 1 **to** *number infected flocks*

do
- *find all susceptible flocks within 0.5km*
- **if** *random num* < *transmission probability*∗ $\left(\left(1 - \frac{dist}{0.5}\right)^2\right)^2$
  - **then**
    - *infect susceptible flock*
    - *note change in flock status*
    - *update farm status to infected*
    - *note date and number of new infections*
    - *set detection time of newly infected farms as above*
    - *set culling dates as above*

As a result of particulate (though not necessarily still infectious) material being detected 500m from poultry houses in the H7N7 outbreak in the Netherlands [D. Alexander, pers. comm.], expert opinion was that spatial (primarily airborne) spread in GB is likely to occur with small probability and only for distances up to a maximum 0.5km [D. Alexander and R. Irvine, pers. comm.]. By assuming that the distance between flocks on the same farm is zero, this allows for between-species spread on multi-species farms and some spatial spread between separate farms. Between-species transmission is important on multi-species sites as it may allow for disease to spread into different industry sectors, which may otherwise not be connected. Based on expert opinion and a density kernel described in [Boender et al., 2007], it is assumed that spatial transmission could occur at a maximum probability of 0.01, up to 0.5km from the infected premises, before detection of disease. This resulted in the probability distribution described in Equation (2) and shown in Additional File 2 Figure S2. The use of this kernel means that the probability of infection via local spread between flocks on the same premises is greater than transmission between flocks on neighbouring premises, as would be likely if, for example, same premises implies greater proximity, or if local spread is actually mediated by human activity,

such as movement of workers on the same premises and poor biosecurity.

$$p\left(transmission|dist\left(km\right)\right)=\begin{cases}0.01\left(\left(1-\frac{dist}{0.5}\right)^{2}\right)^{2} & dist\left(km\right)<0.5km\\ 0\quad otherwise & \end{cases} \tag{2}$$

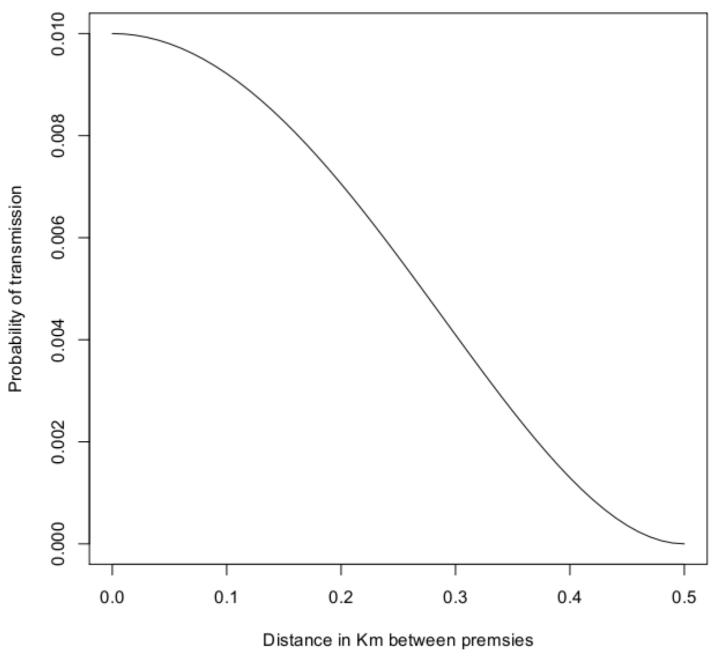

Figure S2. Transmission kernel corresponding to Equation (2), for distances ranging from 0 to 0.5km. The kernel equates to zero for distances larger than 0.5km.

For the list of infected premises, susceptible (ie in this case not culled) premises are found using the 'GBPR_local15_flocks' file (Additional File 2 Table S1). We assume that local spread can occur between flocks on the same premises as well as between premises. There is no within flock spread in the model. Detection and culling dates are set for newly infected premises.

S2.8 Detection and culling

The probability of detection on a daily basis, following infection, will vary according to species, housing type, virus dose and virus strain [Yoon et al., 2005]. Detection (and culling) dates

are set at the same time that a premises becomes infected, dependent on the species infected. The time to detection is given by a triangular distribution, based on mean latent period from the literature [Savill et al., 2008, Sharkey et al., 2008, Stegeman et al., 2004] (Additional File 2 Table S2). For all infected premises, if the infected flock is known to be a duck or goose flock, then the time to detection is increased by 15 days. If multiple flocks are infected, then the time to detection of a premises is equal to the shortest time to detection of all infected flocks on the premises. Premises that are within the PZ or SZ are detected more quickly than those outside these zones (see Table S2). Using the time taken to cull birds in the most recent outbreak of HPAI H5N1 in GB, it is assumed that culling occurs 3 days after detection of an infected premises, reduced to 2 days if the premises is in a SZ and 1 day if it is in a PZ. Once infection has been spread via different routes, detection and culling begins such that for all infected premises, if the detection or culling dates are the same as the current date, the status of the farm is updated (to 2 (detected) or -1 (culled) in 'Farm Inf'). The number of new infections is noted at the end of each day (Additional File 2 Algorithm S1). It is assumed throughout that premises that have been culled are no longer involved in the outbreak. There is no re-housing of culled premises in this model.

The time step is increased by one day and the above processes are repeated on the list of infected premises, up to day 50 and for all transmission parameter combinations.

S2.9 Outputs

Once infection has ceased, the programme records a list of all premises involved in the outbreak. This includes premises that have been infected, detected and culled, as well as premises in the SZ and PZ. The maximum distance between infected premises is calculated as well as mean number of infected premises over all iterations for each combination of transmission probabilities.

Four output files are created.

(i) inf_prems_step.txt: a list of premises infected, the dates that they have been infected, detected and culled, for each simulation.

(ii) PSZSstep.txt: a list of premises included in the PZ and SZ, the dates that they entered the zones for the first time and the date they expect to be removed, for each simulation.

(iii) results_step.txt: the seed farm, the transmission probabilities used, the total epidemic size and the maximum distance between infected premises for each simulation and for each parameter set.

(iv) Output_step.txt: for each parameter set the mean epidemic size, the proportion of seed infections resulting in secondary spread, the largest epidemic simulated (number of premises infected), longest distance between infected premises within largest epidemic, the longest distance between infected premises over all simulations and the number of premises included in the epidemic that is the most widespread are recorded.

Bibliography

[Boender et al., 2007] Boender, G., Hagenaars, T., Bouma, A., Nodelijk, G., Elbers, A., de Jong, M., and van Boven, M. (2007). Risk maps for the spread of highly pathogenic avian influenza in poultry. *PLoS Comput Biol*, 3(4):e71.

[Savill et al., 2008] Savill, N., St Rose, S., and Woolhouse, M. (2008). Detection of mortality clusters associated with highly pathogenic avian influenza in poultry: a theoretical analysis. *Journal of The Royal Society Interface*, 5(29):1409.

[Sharkey et al., 2008] Sharkey, K., Bowers, R., Morgan, K., Robinson, S., and Christley, R. (2008). Epidemiological consequences of an incursion of highly pathogenic H5N1 avian influenza into the British poultry flock. *Proceedings of the Royal Society B: Biological Sciences*, 275(1630):19.

[Stegeman et al., 2004] Stegeman, A., Bouma, A., Elbers, A., de Jong, M., Nodelijk, G., de Klerk, F., Koch, G., and van Boven, M. (2004). Avian influenza A virus (H7N7) epidemic in The Netherlands in 2003: course of the epidemic and effectiveness of control measures. *The Journal of infectious diseases*, 190:2088–2095.

[Yoon et al., 2005] Yoon, H., Park, C., Nam, H., and Wee, S. (2005). Virus spread pattern within infected chicken farms using regression model: the 2003-2004 HPAI epidemic in the Republic of Korea. *Journal of Veterinary Medicine-Berlin-Series B*, 52(10):428.
