# Supplementary material for: The potential spread of highly pathogenic avian influenza virus via dynamic contacts between poultry premises in Great Britain
Source: BMC Vet Res. 2011 Oct 13;7:59. doi: 10.1186/1746-6148-7-59 (PMC3224601; doi:10.1186/1746-6148-7-59)
Supplement: Additional file 3 — Sensitivity to spatial spread. [file 1746-6148-7-59-S3.PDF]

S3 Additional File 3 - Sensitivity to spatial spread

As there is no evidence of airborne spread in any outbreak in GB to inform the model [D. Alexander, pers. comm.], the sensitivity of the simulation model to the assumption that local spread can occur up to 500m was investigated and the results compared for all transmission parameters set to 0.2 (Additional File 3 Table S1). When HPAI was able to spread up to 3km, as seen in parts of the Netherlands in 2002 [Boender et al., 2007], the number of times infection left the network for which movement data were available was more than three times larger for 3km (7.4%) than for 500m (2.4%). There seems to be no significant difference in the number of times infection went beyond the seed premises, for all scenarios. This implies that the probability of infection beyond the seed premises is more heavily dependent on other transmission routes than local spread. An increase of local spread from 500m to 3km was also sufficient to increase both the mean and maximum epidemic size. This highlights the importance of being able to accurately predict the probability of local spread between premises.

Table S1. The impact of assumptions regarding the maximum distance for local spread on the simulated outbreak size for an avian influenza virus in Great Britain.

Local spread limit	Epidemic size		Percentage outbreaks resulting in		Mean number premises	
	Mean	Max	Onward spread	Spread outside system	In PZ	In SZ
0km	2.12	12	21%	0%	124	18
0.5km	1.54	10	20%	2%	78	10
3km	2.53	22	25%	7%	107	15

Bibliography

[Boender et al., 2007] Boender, G., Hagenaars, T., Bouma, A., Nodelijk, G., Elbers, A., de Jong, M., and van Boven, M. (2007). Risk maps for the spread of highly pathogenic avian influenza in poultry. *PLoS Comput Biol*, 3(4):e71.
