# Supplementary material for: The potential spread of highly pathogenic avian influenza virus via dynamic contacts between poultry premises in Great Britain
Source: BMC Vet Res. 2011 Oct 13;7:59. doi: 10.1186/1746-6148-7-59 (PMC3224601; doi:10.1186/1746-6148-7-59)
Supplement: Additional file 4 — Results - supplementary tables. [file 1746-6148-7-59-S4.PDF]

# S4 Additional File 4 - Results - supplementary tables

**Table S1.** Binary logistic regression: secondary spread versus transmission rates for interaction between transmission routes at different levels of transmission.

| Category | Level | odds ratio | Lower 95% | Upper 95% | p-value |
|---|---|---|---|---|---|
| **cc** | 1 | 1.06 | 0.86 | 1.31 | 0.556 |
| | 2 | 1.04 | 0.85 | 1.29 | 0.699 |
| | 3 | 1.11 | 0.90 | 1.37 | 0.325 |
| **own** | 1 | 1.06 | 0.85 | 1.32 | 0.625 |
| | 2 | 1.65 | 1.32 | 2.05 | 0.00 |
| | 3 | 2.06 | 1.66 | 2.56 | 0.00 |
| **sh** | 1 | 0.82 | 0.67 | 1.00 | 0.05 |
| | 2 | 0.89 | 0.73 | 1.08 | 0.243 |
| | 3 | 0.96 | 0.79 | 1.17 | 0.694 |
| **cc*own** | 1*1 | 0.95 | 0.80 | 1.12 | 0.526 |
| | 1*2 | 0.93 | 0.79 | 1.10 | 0.423 |
| | 1*3 | 0.93 | 0.79 | 1.10 | 0.393 |
| | 2*1 | 0.98 | 0.83 | 1.16 | 0.808 |
| | 2*2 | 0.95 | 0.80 | 1.13 | 0.573 |
| | 2*3 | 0.94 | 0.80 | 1.11 | 0.490 |
| | 3*1 | 0.93 | 0.78 | 1.10 | 0.396 |
| | 3*2 | 0.88 | 0.74 | 1.04 | 0.126 |
| | 3*3 | 0.87 | 0.74 | 1.03 | 0.100 |
| **cc*sh** | 1*1 | 0.97 | 0.85 | 1.12 | 0.688 |
| | 1*2 | 0.99 | 0.86 | 1.14 | 0.916 |
| | 1*3 | 0.97 | 0.84 | 1.11 | 0.651 |
| | 2*1 | 0.99 | 0.86 | 1.14 | 0.870 |
| | 2*2 | 1.00 | 0.87 | 1.15 | 0.967 |
| | 2*3 | 0.96 | 0.83 | 1.10 | 0.534 |
| | 3*1 | 0.98 | 0.86 | 1.13 | 0.831 |
| | 3*2 | 1.01 | 0.88 | 1.16 | 0.871 |
| | 3*3 | 0.97 | 0.84 | 1.11 | 0.635 |
| **own*sh** | 1*1 | 1.28 | 1.09 | 1.50 | 0.003 |
| | 1*2 | 1.25 | 1.07 | 1.47 | 0.006 |
| | 1*3 | 1.25 | 1.07 | 1.47 | 0.006 |
| | 2*1 | 1.23 | 1.05 | 1.44 | 0.012 |
| | 2*2 | 1.16 | 0.99 | 1.36 | 0.061 |
| | 2*3 | 1.15 | 0.98 | 1.34 | 0.089 |
| | 3*1 | 1.25 | 1.07 | 1.47 | 0.005 |
| | 3*2 | 1.19 | 1.01 | 1.39 | 0.033 |
| | 3*3 | 1.16 | 0.99 | 1.36 | 0.058 |

Level 1 = low transmission rate 0 - 0.06, level 2 = medium transmission rate 0.07 - 0.13, level 3 = high transmission rate 0.14 - 0.2. cc= catching company, sh = slaughterhouse, own = company personnel.

**Table S2.** Binary logistic regression: small outbreaks versus catching company transmission rates.

| Transmission rate | Odds ratio | Lower 95% | Upper 95% | p-value |
|---|---|---|---|---|
| 0.001 | 0.97 | 0.93 | 1.00 | 0.064 |
| 0.01 | 1.00 | 0.97 | 1.04 | 0.820 |
| 0.02 | 0.95 | 0.92 | 0.98 | 0.005 |
| 0.03 | 0.97 | 0.94 | 1.01 | 0.153 |
| 0.04 | 1.02 | 0.98 | 1.06 | 0.303 |
| 0.05 | 0.96 | 0.93 | 0.99 | 0.024 |
| 0.06 | 0.96 | 0.93 | 1.00 | 0.044 |
| 0.07 | 0.99 | 0.95 | 1.02 | 0.451 |
| 0.08 | 0.96 | 0.93 | 1.00 | 0.037 |
| 0.09 | 0.95 | 0.92 | 0.99 | 0.011 |
| 0.10 | 0.97 | 0.94 | 1.01 | 0.109 |
| 0.11 | 0.98 | 0.94 | 1.01 | 0.230 |
| 0.12 | 0.99 | 0.96 | 1.03 | 0.697 |
| 0.13 | 1.00 | 0.97 | 1.04 | 0.971 |
| 0.14 | 0.98 | 0.94 | 1.01 | 0.172 |
| 0.15 | 0.98 | 0.94 | 1.01 | 0.241 |
| 0.16 | 0.96 | 0.92 | 0.99 | 0.016 |
| 0.17 | 1.00 | 0.96 | 1.03 | 0.807 |
| 0.18 | 0.98 | 0.95 | 1.02 | 0.313 |
| 0.19 | 0.96 | 0.93 | 1.00 | 0.045 |
| 0.20 | 0.98 | 0.95 | 1.02 | 0.373 |

**Table S3.** Binary logistic regression: small outbreaks versus owner transmission rates.

| Transmission rate | Odds ratio | Lower 95% | Upper 95% | p-value |
|---|---|---|---|---|
| 0.001 | 0.98 | 0.94 | 1.03 | 0.488 |
| 0.01 | 1.09 | 1.04 | 1.14 | 0.000 |
| 0.02 | 1.19 | 1.14 | 1.25 | 0.000 |
| 0.03 | 1.27 | 1.22 | 1.33 | 0.000 |
| 0.04 | 1.38 | 1.33 | 1.44 | 0.000 |
| 0.05 | 1.42 | 1.37 | 1.48 | 0.000 |
| 0.06 | 1.47 | 1.41 | 1.54 | 0.000 |
| 0.07 | 1.53 | 1.47 | 1.59 | 0.000 |
| 0.08 | 1.66 | 1.59 | 1.73 | 0.000 |
| 0.09 | 1.74 | 1.67 | 1.81 | 0.000 |
| 0.10 | 1.76 | 1.69 | 1.83 | 0.000 |
| 0.11 | 1.86 | 1.79 | 1.94 | 0.000 |
| 0.12 | 1.97 | 1.89 | 2.05 | 0.000 |
| 0.13 | 1.94 | 1.87 | 2.02 | 0.000 |
| 0.14 | 2.11 | 2.03 | 2.19 | 0.000 |
| 0.15 | 2.09 | 2.01 | 2.17 | 0.000 |
| 0.16 | 2.19 | 2.11 | 2.28 | 0.000 |
| 0.17 | 2.24 | 2.16 | 2.33 | 0.000 |
| 0.18 | 2.34 | 2.25 | 2.43 | 0.000 |
| 0.19 | 2.38 | 2.29 | 2.48 | 0.000 |
| 0.20 | 2.38 | 2.29 | 2.48 | 0.000 |

**Table S4.** Binary logistic regression: small outbreaks versus slaughterhouse transmission rates.

| Transmission rate | Odds ratio | Lower 95% | Upper 95% | p-value |
|---|---|---|---|---|
| 0.001 | 0.97 | 0.94 | 1.01 | 0.101 |
| 0.01 | 0.99 | 0.95 | 1.02 | 0.446 |
| 0.02 | 0.99 | 0.96 | 1.03 | 0.623 |
| 0.03 | 1.00 | 0.96 | 1.03 | 0.883 |
| 0.04 | 1.00 | 0.96 | 1.03 | 0.846 |
| 0.05 | 1.00 | 0.96 | 1.03 | 0.897 |
| 0.06 | 1.04 | 1.00 | 1.08 | 0.037 |
| 0.07 | 1.03 | 1.00 | 1.07 | 0.068 |
| 0.08 | 1.04 | 1.01 | 1.08 | 0.022 |
| 0.09 | 1.04 | 1.01 | 1.08 | 0.021 |
| 0.10 | 1.05 | 1.01 | 1.08 | 0.014 |
| 0.11 | 1.07 | 1.03 | 1.11 | 0.000 |
| 0.12 | 1.05 | 1.01 | 1.09 | 0.010 |
| 0.13 | 1.09 | 1.05 | 1.13 | 0.000 |
| 0.14 | 1.08 | 1.04 | 1.12 | 0.000 |
| 0.15 | 1.09 | 1.05 | 1.13 | 0.000 |
| 0.16 | 1.07 | 1.04 | 1.11 | 0.000 |
| 0.17 | 1.08 | 1.04 | 1.12 | 0.000 |
| 0.18 | 1.09 | 1.05 | 1.13 | 0.000 |
| 0.19 | 1.09 | 1.05 | 1.13 | 0.000 |
| 0.20 | 1.10 | 1.06 | 1.14 | 0.000 |

**Table S5.** Binary logistic regression: large outbreaks versus catching company transmission rates.

| Transmission rate | Odds ratio | Lower 95% | Upper 95% | p-value |
|---|---|---|---|---|
| 0.001 | 1.00 | 0.43 | 2.31 | 0.999 |
| 0.01 | 1.09 | 0.48 | 2.47 | 0.836 |
| 0.02 | 1.09 | 0.48 | 2.47 | 0.836 |
| 0.03 | 0.54 | 0.20 | 1.47 | 0.232 |
| 0.04 | 1.00 | 0.43 | 2.31 | 0.999 |
| 0.05 | 0.91 | 0.39 | 2.14 | 0.826 |
| 0.06 | 0.91 | 0.39 | 2.14 | 0.826 |
| 0.07 | 1.00 | 0.43 | 2.31 | 0.999 |
| 0.08 | 1.09 | 0.48 | 2.47 | 0.836 |
| 0.09 | 1.00 | 0.43 | 2.31 | 0.999 |
| 0.10 | 1.64 | 0.77 | 3.46 | 0.199 |
| 0.11 | 1.00 | 0.43 | 2.31 | 0.999 |
| 0.12 | 0.73 | 0.29 | 1.81 | 0.492 |
| 0.13 | 1.82 | 0.87 | 3.79 | 0.112 |
| 0.14 | 1.45 | 0.67 | 3.13 | 0.339 |
| 0.15 | 1.64 | 0.77 | 3.46 | 0.199 |
| 0.16 | 1.36 | 0.63 | 2.97 | 0.435 |
| 0.17 | 1.82 | 0.87 | 3.79 | 0.112 |
| 0.18 | 1.00 | 0.43 | 2.31 | 0.999 |
| 0.19 | 1.18 | 0.53 | 2.64 | 0.685 |
| 0.20 | 0.73 | 0.29 | 1.81 | 0.492 |

**Table S6.** Binary logistic regression: large outbreaks versus owner transmission rates.

| **Transmission rate** | **Odds ratio** | **Lower 95%** | **Upper 95%** | **p-value** |
|---|---|---|---|---|
| 0.001 | 1.00 | 0.45 | 2.23 | 0.999 |
| 0.01 | 0.58 | 0.23 | 1.48 | 0.256 |
| 0.02 | 0.58 | 0.23 | 1.48 | 0.256 |
| 0.03 | 0.67 | 0.27 | 1.63 | 0.374 |
| 0.04 | 1.08 | 0.49 | 2.37 | 0.843 |
| 0.05 | 0.92 | 0.40 | 2.08 | 0.834 |
| 0.06 | 0.92 | 0.40 | 2.08 | 0.834 |
| 0.07 | 0.83 | 0.36 | 1.93 | 0.669 |
| 0.08 | 1.00 | 0.45 | 2.23 | 0.999 |
| 0.09 | 1.33 | 0.63 | 2.82 | 0.452 |
| 0.10 | 1.25 | 0.58 | 2.67 | 0.565 |
| 0.11 | 1.00 | 0.45 | 2.23 | 0.999 |
| 0.12 | 1.58 | 0.77 | 3.26 | 0.213 |
| 0.13 | 1.17 | 0.54 | 2.52 | 0.696 |
| 0.14 | 0.83 | 0.36 | 1.93 | 0.669 |
| 0.15 | 1.83 | 0.91 | 3.70 | 0.091 |
| 0.16 | 0.92 | 0.40 | 2.08 | 0.834 |
| 0.17 | 0.92 | 0.40 | 2.08 | 0.834 |
| 0.18 | 1.08 | 0.49 | 2.37 | 0.843 |
| 0.19 | 1.08 | 0.49 | 2.37 | 0.843 |
| 0.20 | 1.33 | 0.63 | 2.82 | 0.452 |

**Table S7.** Binary logistic regression: large outbreaks versus slaughterhouse transmission rates.

| **Transmission rate** | **Odds ratio** | **Lower 95%** | **Upper 95%** | **p-value** |
|---|---|---|---|---|
| 0.001 | 0.00 | 0.00 | na | 0.996 |
| 0.01 | 0.50 | 0.09 | 2.73 | 0.422 |
| 0.02 | 0.00 | 0.00 | na | 0.996 |
| 0.03 | 0.25 | 0.03 | 2.23 | 0.214 |
| 0.04 | 0.25 | 0.03 | 2.23 | 0.214 |
| 0.05 | 2.00 | 0.60 | 6.63 | 0.259 |
| 0.06 | 1.25 | 0.34 | 4.65 | 0.741 |
| 0.07 | 1.00 | 0.25 | 3.99 | 0.998 |
| 0.08 | 2.50 | 0.78 | 7.96 | 0.122 |
| 0.09 | 2.25 | 0.69 | 7.29 | 0.178 |
| 0.10 | 2.25 | 0.69 | 7.29 | 0.178 |
| 0.11 | 3.00 | 0.97 | 9.29 | 0.057 |
| 0.12 | 3.49 | 1.15 | 10.62 | 0.027 |
| 0.13 | 3.00 | 0.97 | 9.29 | 0.057 |
| 0.14 | 3.25 | 1.06 | 9.95 | 0.04 |
| 0.15 | 5.49 | 1.89 | 15.94 | 0.002 |
| 0.16 | 6.49 | 2.27 | 18.60 | 0.000 |
| 0.17 | 6.99 | 2.45 | 19.94 | 0.000 |
| 0.18 | 6.24 | 2.17 | 17.94 | 0.001 |
| 0.19 | 10.24 | 3.67 | 28.59 | 0.000 |
| 0.20 | 7.24 | 2.55 | 20.60 | 0.000 |
