# Supplementary material for: The potential spread of highly pathogenic avian influenza virus via dynamic contacts between poultry premises in Great Britain
Source: BMC Vet Res. 2011 Oct 13;7:59. doi: 10.1186/1746-6148-7-59 (PMC3224601; doi:10.1186/1746-6148-7-59)
Supplement: Additional file 5 — Maximum distance between infected premises. [file 1746-6148-7-59-S5.PDF]

S5 Additional File 5 - Maximum distance between infected premises.

The results of the maximum distance between infected premises are displayed in Additional File 5 Figure S1

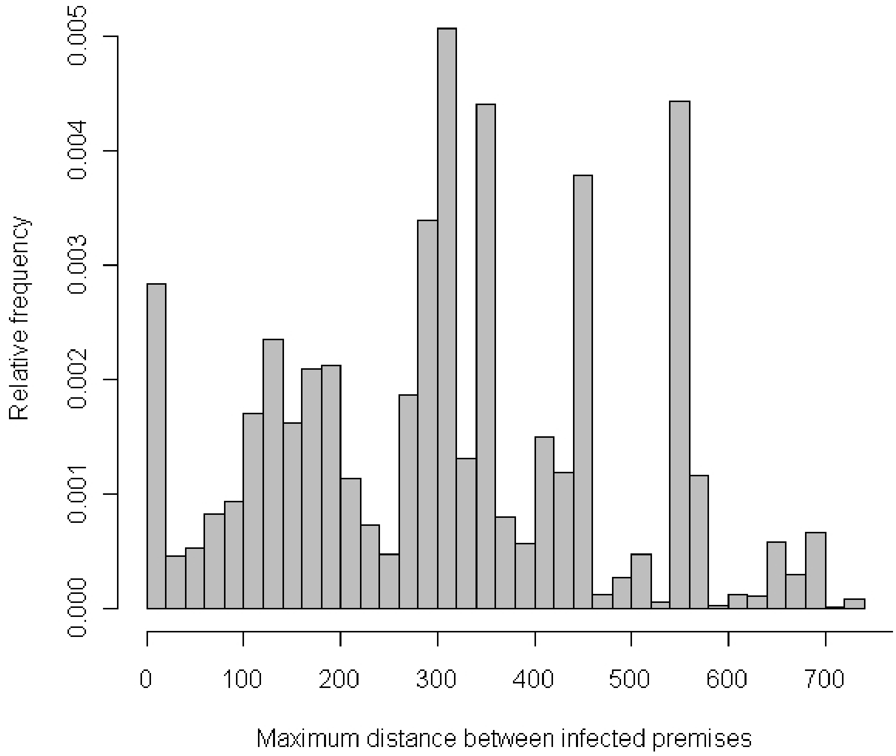

Figure S1. Histogram of maximum distance between infected premises for all outbreaks that spread beyond the seed premises
